# Dietary Plant and Animal Protein Sources Oppositely Modulate Fecal *Bilophila* and *Lachnoclostridium* in Vegetarians and Omnivores

**DOI:** 10.1128/spectrum.02047-21

**Published:** 2022-03-14

**Authors:** Ya-Ting Wu, Shou-Ju Shen, Kuan-Fu Liao, Ching-Ying Huang

**Affiliations:** a Department of Food Science and Biotechnology, National Chung Hsing University, Taichung, Taiwan; b Department of Family Medicine, Taichung Tzu Chi Hospital, Buddhist Tzu Chi Medical Foundation, Taichung, Taiwan; c Taichung Tzu Chi Nursing Home, Taichung, Taiwan; d St. Martin De Porres Hospital, Chiayi City, Taiwan; e Department of Internal Medicine, Taichung Tzu Chi Hospital, Buddhist Tzu Chi Medical Foundation, Taichung, Taiwan; Huazhong University of Science and Technology

**Keywords:** gut microbiome, pathobionts, diet, metabolic disease, colorectal cancer, inflammatory bowel disease

## Abstract

The food we eat not only nourishes our bodies but also provides nutrients to the bacteria living in our guts. Gut bacterial communities are known to be affected by many factors, including diet and bowel cleansing, but the impacts of vegetarian and omnivore diets on fecal bacterial composition are still uncertain. In this study, we analyzed the bacterial compositions of fecal samples from vegetarians and omnivores 5 to 7 days after bowel cleansing, and we correlated specific dietary constituents with the relative abundances of specialized fecal bacteria. A total of 46 participants (23 vegetarians and 23 omnivores) were recruited. All participants underwent standard bowel cleansing before colonoscopy screening. Fecal samples were collected from each participant 5 to 7 days after bowel cleansing, and the fecal microbiota compositions were analyzed with next-generation sequencing. Sixteen participants also provided an image-based dietary record for nutritional assessment. No major differences between dietary groups were observed in terms of fecal bacterial richness, alpha diversity, or beta diversity. A minority of potential pathobionts tended to be elevated in omnivores compared to vegetarians, whereas potential probiotic species tended to be higher in the vegetarians. Detailed dietary assessments further revealed that the plant- and animal-derived proteins may oppositely modulate the relative abundances of pathobionts Bilophila and Lachnoclostridium. However, these results were not statistically significant after multiple-comparison correction. These results suggest that specialized probiotic and pathobiont microbiota constituents are sensitive to the plant- or animal-derived dietary components ingested by vegetarians and omnivores after bowel cleansing.

**IMPORTANCE** Dietary pattern and food choice are associated with expansion of gut pathobionts and risk for metabolic and colonic disease. However, the effects of dietary interventions on intestinal microbiota remain unclear. After bowel cleansing, potential pathobionts and probiotic bacteria were increased in omnivores and vegetarians, respectively. The pathobionts Bilophila and Lachnoclostridium were oppositely modulated by dietary animal and plant protein. From a clinical perspective, fecal pathobionts that may indicate risk for metabolic and colonic disease can potentially be modulated with dietary interventions.

## INTRODUCTION

Dietary patterns may exert distinct nutritional effects on both the host and their gut bacteria (1). It is well known that the intestinal and fecal microbial composition responds continually and rapidly to many factors, including diet (1–3), environmental exposures (4), the host genome (5, 6), lifestyle (3, 7), hygiene (8, 9), and use of antibiotics (10, 11). Furthermore, both the intestinal microbiota *per se* and microbiota-generated metabolites have been shown to mediate interactions between diet and disease (1, 12–14). While vegetarian diets are often perceived by the public as being healthier overall than omnivore diets, the effects of vegetarian diets on intestinal microbiota and disease remain to be fully elucidated (15–17).

Some positive effects of vegetarian diets have been previously reported (1, 18). Early research on vegetarian diets focused on the direct effects of certain dietary components on physiological functions and pathological disease progression, especially with regard to high fiber, potassium and magnesium contents, antioxidant properties of vitamins, and the protective capacities of numerous phytochemicals in plant-based foods (18, 19). Additionally, the roles of certain macronutrients in vegetarian diets, such as plant-based protein, have been associated with longevity and lower risks of mortality from obesity (20), type 2 diabetes (T2D) (21), cardiovascular disease (CVD) (22), and cancer (15, 22). Over the last decade, major advancements have been made in our understanding of intestinal bacteria, and many effects of vegetarian diets on gut microbiota and their metabolites have been reported (1, 16, 17). Furthermore, it has been suggested that there is a positive association between animal protein intake and risks of CVD (23) and colorectal cancer (CRC) (24); these increased risks may be the consequence of high levels of intestinal bacteria-produced hydrogen sulfide (H_2_S) and trimethylamine-*N*-oxide (TMAO). However, contradictory results and inconsistent outcomes have been reported in comparisons of vegetarian and omnivore dietary effects on intestinal flora (16, 25). These conflicting results may be attributable to many factors, such as the length of time for dietary intervention, geographical variations, interindividual variability, or the methods applied for feces collection. At present, it therefore remains unclear precisely how intestinal microbiota respond to dietary interventions.

The profile of human gut microbiota is shaped from birth in a process that continues throughout the individual’s life span. The microbial composition in the colon is dynamic and changes rapidly due to many influences (26), including bowel cleansing prior to colonic endoscopic examination, bowel surgery, and fecal microbiota transplantation (27–29). Additionally, different bowel cleansing methods have been shown to influence the fecal microbiota to various degrees (27–30). Previous studies showed that bowel cleansing with polyethylene glycol (PEG) results in an immediate decrease of total microbial load and alters biodiversity; the microbiota are largely restored within 14 days to 1 month, accompanied by slight changes in composition (27, 29). Notably, a small-scale study suggested that only a minority of subjects are susceptible to PEG-induced perturbations (28). Although most studies on vegetarian diets suggest some beneficial effects on the intestinal microbiota (1, 31), the results do not indicate with any certainty which specific bacterial populations are subject to change (16, 25) or how bowel cleansing might affect the intervention. It is reasonable to expect that bowel cleansing prior to collection of fecal samples may reveal more precise associations between dietary pattern and intestinal microbiota composition, as the fecal bacterial changes would not be masked by historical population dynamics. Moreover, a recent report suggested that a constitutive bowel enema during vegetarian dietary intervention may produce a more sustained beneficial effect on the gut microbiota in patients with Parkinson’s disease (32). Thus, bowel cleansing may be useful as a clinical means to accelerate alterations in intestinal microbiota. Nevertheless, whether dietary composition can impact the reestablishment of gut microbiota after bowel cleansing remains unknown.

In the current study, fecal samples were collected from volunteers on vegetarian and omnivore diets 5 to 7 days after bowel cleansing for CRC screening. The compositions of fecal microbiota were determined for each participant. Moreover, detailed assessments of diet were performed on a subset of 16 participants. We then compared the bacterial compositions and relative bacterial abundances between diet groups, and we further evaluated correlations between specialized bacteria and specific dietary constituents.

## RESULTS

**FIG 1:**
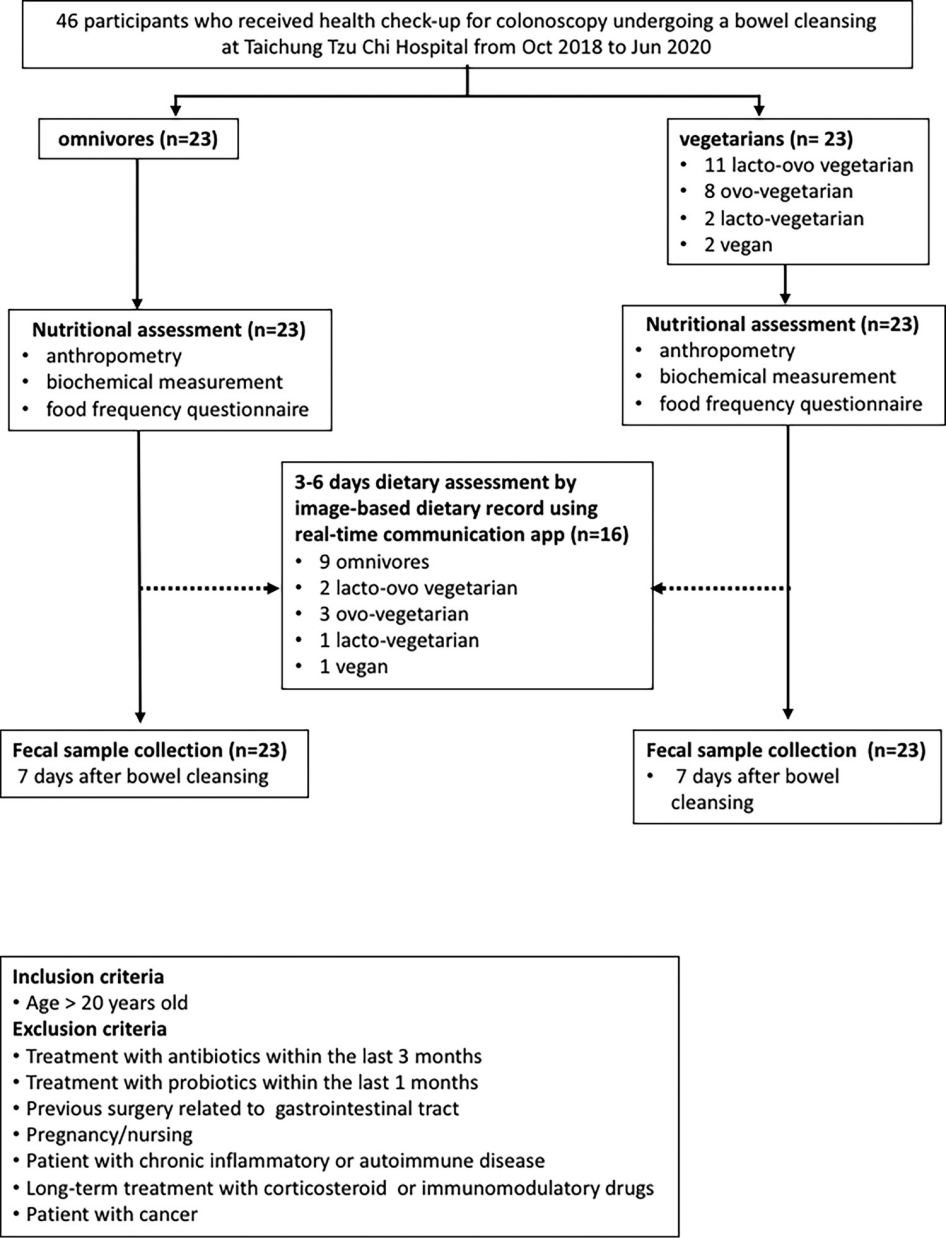
Flow diagram of participants included in this study.

**FIG 2:**
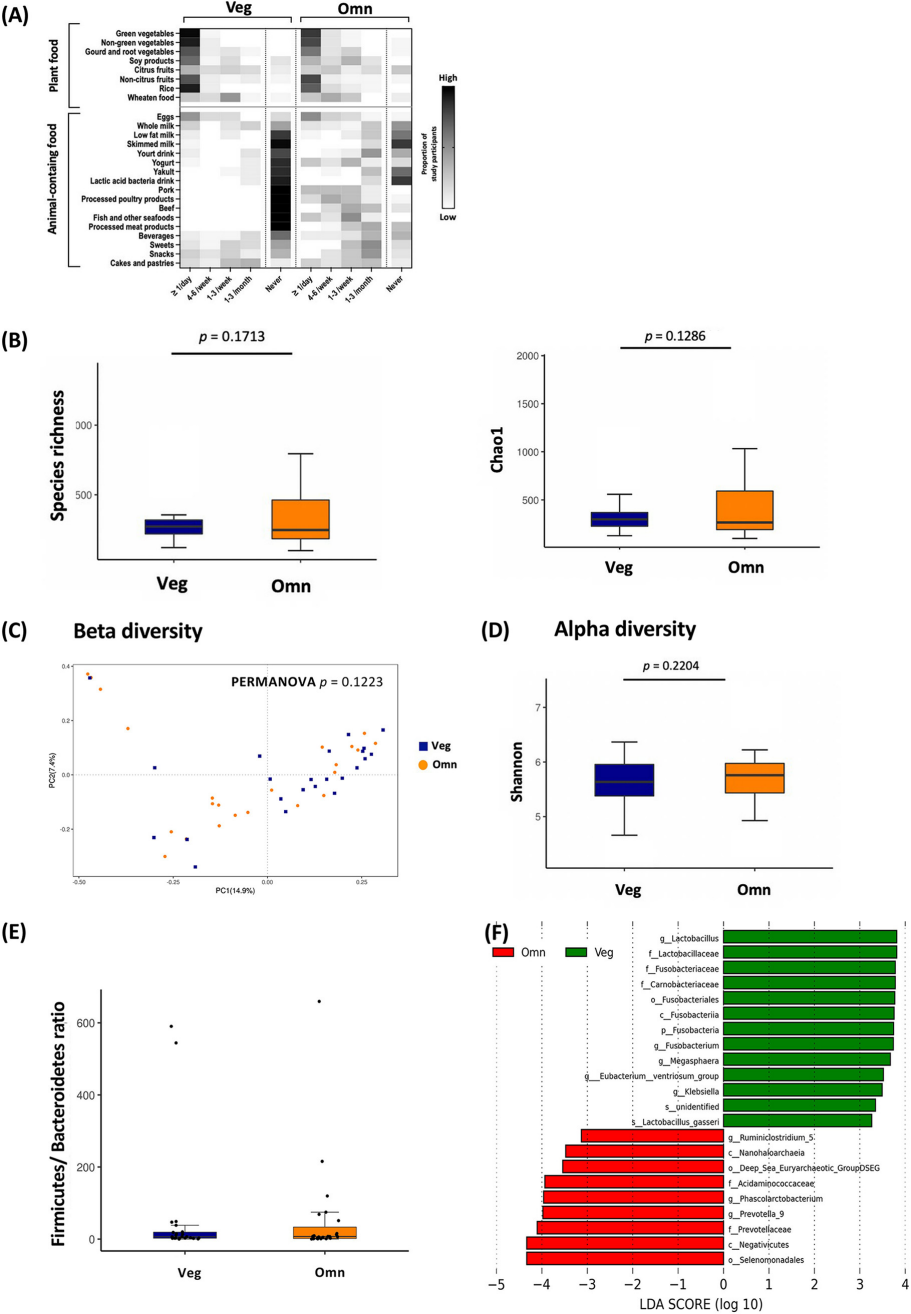
Vegetarians and omnivores displayed no major differences in fecal microbiota composition after bowel cleansing. (A) Heatmap of food intake frequency in vegetarians (Veg) and omnivores (Omn) based on the food frequency questionnaire (FFQ). Each heatmap square indicates the proportion of study participants consuming a specific food with the indicated frequency. Darker shade indicates more people consumed that category of food. On average, vegetarian diets included no meat, low dairy/eggs, and high plant-based food. Rows, food category; columns, intake frequency. (B to E) No significant differences between diet groups were found in fecal bacterial species richness (B), beta diversity by principal coordinate analysis calculated using Bray-Curtis distance (PERMANOVA, *P = *0.1223) (C), alpha diversity (D), or Firmicutes/Bacteroidetes ratio (E). (F) Histogram of the linear discriminant analysis (LDA) score demonstrated different taxa in fecal microbiota between Veg (top) and Omn (bottom).

By analyzing the microbiota in fecal samples from participants, we found that the vegetarian diet contributed to only slight differences in fecal bacterial richness and diversity. A total of 23 vegetarian and 23 omnivore participants were enrolled in this study, and 16 participants completed an additional image-based dietary assessment. A flowchart summarizing the participants and study design is shown in Fig. 1. Food frequency questionnaire (FFQ)-based long-term dietary assessments for all participants are summarized in Fig. 2A. Vegetarians consumed mostly plant-based foods, with a small proportion of eggs and dairy products. The omnivores consumed various animal-derived foods with modest amounts of plant-based food. It is worth noting that vegetarians had a higher intake of soy-based foods than omnivores. A comparison of biochemical values revealed significantly higher levels of total cholesterol in omnivores (Table 1).

**TABLE 1:** Summary of participants’ demographic and metabolic characteristics

Characteristic	Value [mean ± SD or no. (%)] for:		*P* value^*a*^	*q* value
	Vegetarians (*n* = 23)	Omnivores (*n* = 23)		
Age (yr)	58.83 ± 17.68	51.04 ± 4.95		
<65	16 (73.9)	22 (95.7)		
≥65	6 (26.1)	1 (4.3)		
Gender				
Male	10 (43.5)	10 (43.5)		
Female	13 (56.5)	13 (56.5)		
Body mass index (kg/m^2^)	23.18 ± 0.56	23.52 ± 2.24	0.70	0.94
Body fat (%)	29.53 ± 11.67	30.47 ± 12.02	0.62	1.00
Male	23.46 ± 1.06	26.33 ± 0.78	0.17	1.00
Female	34.20 ± 4.95	36.66 ± 11.88	0.76	0.92
Blood pressure (mm Hg)				
Systolic	124.09 ± 4.95	121.39 ± 14.85	0.45	0.95
Diastolic	75.17 ± 19.80	73.09 ± 9.90	0.47	0.85

Plasma biochemical data^*b*^				
Fasting blood glucose (mg/dL)	100.57 ± 12.73	95.00 ± 0.71	0.21	1.00
HbA1c (%)	5.79 ± 0.71	5.48 ± 0.21	0.07	1.00
Triglyceride (mg/dL)	119.57 ± 44.55	144.22 ± 69.30	0.25	1.00
Total cholesterol (mg/dL)	169.22 ± 36.06	202.17 ± 28.99	0.08*	0.23
LDL-C (mg/dL)	107.13 ± 25.46	127.17 ± 17.68	0.08	1.00
AST (IU/L)	23.61 ± 10.61	21.30 ± 3.54	0.31	0.91
ALT (IU/L)	27.09 ± 0.71	28.87 ± 2.12	0.73	0.92
BUN (mg/dL)	9.04 ± 2.12	9.43 ± 4.95	0.64	1.00
Creatinine	0.80 ± 0.14	0.79 ± 0.35	0.86	0.98
Uric acid (mg/dL)	4.98 ± 0.71	5.07 ± 0.92	0.77	0.96

a The *P* value is the comparison between vegetarian and omnivore volunteers using Student’s *t* test. *, *P *< 0.05.

b HbA1c, glycated hemoglobin; LDL-C, low-density lipoprotein-cholesterol; AST, aspartate aminotransferase; ALT, alanine aminotransferase; BUN, blood urea nitrogen.

The fecal bacterial profiles of the vegetarian and omnivore participants did not differ considerably in terms of species richness (Fig. 2B), beta diversity (Fig. 2C), alpha diversity (Fig. 2D), or Firmicutes/Bacteroidetes ratio (Fig. 2E). These results are similar to previous findings (12, 23, 33). In total, we identified 22 fecal bacterial taxa that were mostly distinct between vegetarians and omnivores according to the linear discriminant analysis (LDA) score (LDA score > 3.0) (Fig. 2F; Table S6 in the supplemental material). However, there were no significant results among them after multiple-comparison correction.

### Relative abundances of specific fecal bacteria differ between vegetarians and omnivores.

**FIG 3:**
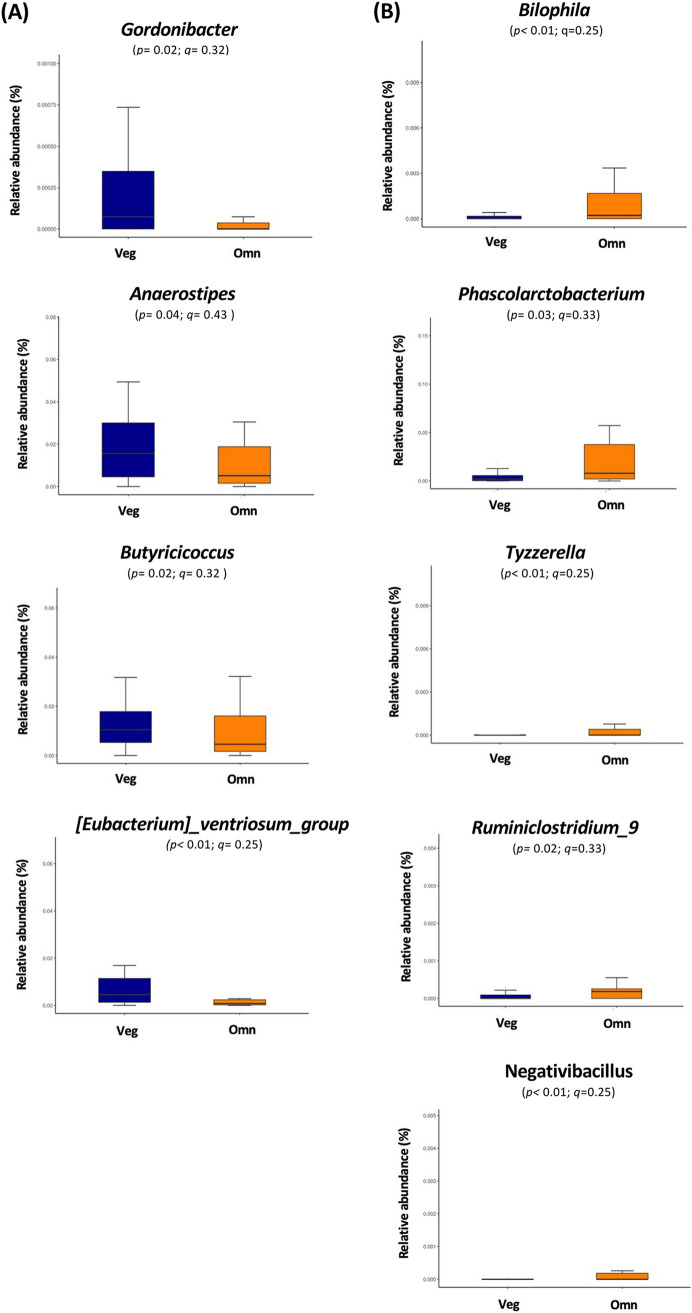
MetagenomeSeq revealed differences in certain bacteria between vegetarians and omnivores after bowel cleansing. MetagenomeSeq identified the relative abundances of fecal bacterial genus levels in vegetarians (Veg) and omnivores (Omn) (A), including Gordonibacter, Anaerostipes, Butyricicoccus, and *ventriosum*_group. The analysis also identified genera that were higher in Omn than Veg (B), including Bilophila, Phascolarctobacterium, Tyzzerella, Ruminiclostridium_9, and Negativibacillus. Adjusted *P* values are expressed as *q* values.

Next, we made a closer examination of the microbiota profiles of all participants by using metagenomeSeq. The differences between vegetarians and omnivores in the relative abundances of specific bacteria at the genus level after bowel cleansing were not statistically significant. The relative abundances of fecal Gordonibacter, Anaerostipes, Butyricicoccus, and (Eubacterium) *ventriosum*_group tended to be higher in the vegetarians than in omnivores (Fig. 3A). In contrast, the relative abundances of fecal Bilophila, Phascolarctobacterium, Tyzzerella, Ruminiclostridium_9, and Negativibacillus tended to be higher in omnivores than in vegetarians (Fig. 3B). However, there were no significant differences after correction for multiple comparisons.

### Relative abundances of particular fecal bacteria are sensitive to the levels of plant- or animal-derived proteins in the diet.

**FIG 4:**
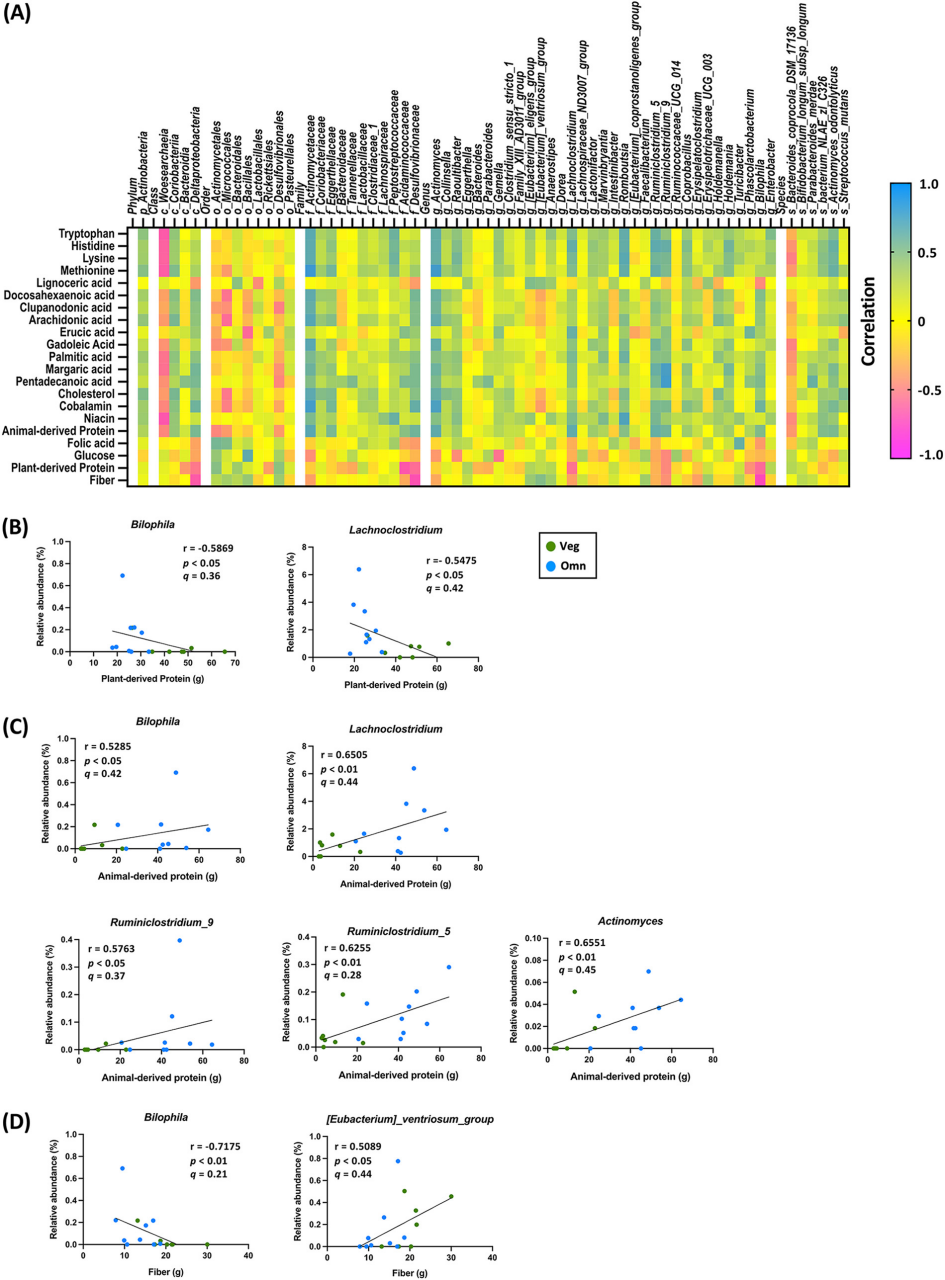
Certain fecal bacteria were sensitive to distinct dietary nutrients and displayed opposite correlations with plant and animal protein after bowel cleansing. A total of 16 participants, including 7 vegetarians (Veg) and 9 omnivores (Omn), provided image-based dietary records. For nutrients with intakes that were significantly different between the two dietary groups, correlation analyses with fecal bacterial abundances were performed. (A) Heatmap summarizes Spearman correlations for pairwise comparisons of nutrient intake (rows) and bacterial taxa (columns). (B) Correlations between plant-derived protein and relative abundances of Bilophila and Lachnoclostridium. (C) Correlations between animal-derived protein and relative abundances of Bilophila, Lachnoclostridium, Actinomyces, Ruminiclostridium_5, and Ruminiclostridium_9. (D) Correlations between dietary fiber and relative abundances of Bilophila and (Eubacterium) *ventriosum*_group genera.

A total of 16 participants (7 vegetarians and 9 omnivores) further completed an average 4-day image-based dietary assessment after bowel cleansing. We analyzed the daily intakes of major nutrients, monosaccharides, fatty acids, amino acids, vitamins, and minerals (Tables S1 to S5). Among these nutrients, intakes of fiber, plant-derived protein, glucose, folic acid, and lignoceric acid were significantly higher in vegetarians than in omnivores. The intakes of animal-derived protein, cholesterol, niacin, cobalamin, several fatty acids, and amino acids were higher for omnivores than for vegetarians. We further analyzed the correlations between nutrients that differed between groups and the relative abundances of fecal bacteria. The results are shown in a heatmap (Fig. 4A); Spearman correlation coefficients, original *P* values, and adjusted *P* values (*q* values) are shown in Table S7. The multiple-comparison correction was performed separately for different taxonomic ranks.

### Fecal abundances of *Bilophila* and *Lachnoclostridium* are correlated with dietary plant and animal proteins.

The detailed dietary assessments revealed no difference in total protein intake. Expectedly, however, the vegetarians had a significantly higher intake of plant protein, while the omnivores had a significantly higher intake of animal protein (Table S1).

To investigate the contributions of distinct protein sources to the bacterial signature, we first correlated dietary plant- and animal-derived protein with bacterial taxa in vegetarians and omnivores. The relative abundances of fecal Bilophila and Lachnoclostridium were inversely correlated with dietary plant protein intakes (*r* = −0.5869 and −0.5475, respectively; *P < *0.05; *q* not significant) (Fig. 4B) and positively correlated with dietary animal protein intakes (*r* = 0.5285, *P < *0.05 and *r* = 0.6505, *P < *0.01, respectively; *q* not significant) (Fig. 4C). In addition, the relative abundance of fecal Bilophila was also negatively correlated with dietary fiber (*r* = −0.7175, *q *< 0.05) (Fig. 4D). The abundances of other fecal bacteria, including Actinomyces (*r* = 0.6551, *q *< 0.05), Ruminiclostridium_5 (*r* = 0.6255, *P < *0.05, *q* not significant), and Ruminiclostridium_9 (*r* = 0.6777, *q *< 0.05), were positively correlated with dietary animal protein (Fig. 4C). (*Eubacterium*) *ventriosum*_group was positively correlated with dietary fiber (*r* = 0.5089, *q* not significant) (Fig. 4D).

## DISCUSSION

In this study, we collected fecal samples from vegetarians and omnivores 5 to 7 days after bowel cleansing. We found that some potential pathobiont species were relatively higher in omnivores, while some potential probiotic species were higher in vegetarians. Moreover, the pathobiont species Bilophila and Lachnoclostridium were positively correlated with dietary animal protein and negatively correlated with dietary plant protein sources. Changes in an individual’s fecal bacterial composition before and after bowel cleansing were not evaluated due to a lack of precleansing samples. Furthermore, the small sample size in this study led to a lack of significant differences according to adjusted *P* (*q*) value. Nevertheless, our results implied that a minority of fecal bacteria might be differentially modulated by vegetarian and omnivore diets, since no significant differences in major microbiota composition were found between groups. The health effects of these specific changes remain unknown and warrant further study. Understanding how distinct fecal bacteria differ in individuals with different dietary intakes after bowel cleansing will be important for the clinical application of personalized nutrition in the era of individualized medicine.

In general, vegetarian diets are purported to be rich in fiber and low in protein and fat. We conducted a thorough dietary assessment for 16 participants and showed that the fiber intake of vegetarians was significantly higher than that of omnivores. However, no significant differences were observed in total protein or fat intake between groups. Compared to vegetarian diets in Western countries, vegetarians in Taiwan have been shown to ingest more plant-derived protein from soy products (34, 35). In the bacterial analysis, we observed no significant changes in species richness, beta diversity, alpha diversity, or Firmicutes/Bacteroidetes ratio. These findings are compatible with previous studies (12, 17, 23, 33) and underscore the importance of performing detailed analysis of individual diet compositions (e.g., image-based records) when studying the relationship between diet and intestinal bacteria. Furthermore, the effects of bowel cleansing should be taken into account when assessing dietary effects on intestinal microbiota. In one recent small cohort study, the gut microbiomes of healthy subjects and patients with Parkinson’s disease were evaluated after a combination of bowel cleansing (oil enema for 8 consecutive days) and dietary intervention (ovo-lacto vegetarian diet plus short-chain fatty acid for 14 days). Remarkably, the bowel cleansing was associated with a positive effect on the gut microbiome and decreased drug dosage in a 1-year follow-up compared to dietary intervention alone (32). While our study would have benefited from larger sample sizes to generate better evidence, the results still provide novel information about how combinations of dietary intervention and bowel cleansing may be applied in the clinic.

Our metagenomeSeq analysis revealed that the fecal loads of Gordonibacter, Anaerostipes, Butyricicoccus, and *ventriosum*_group in vegetarians tended to be higher than those in omnivores. It has been reported that Butyricicoccus pullicaecorum can metabolize ellagitannin and ellagic acid into urolithins in the intestine, which may have positive effects on health (36–38). Additionally, low relative fecal abundances of butyrate-producing Anaerostipes and *ventriosum*_group have been reported in patients with CRC (39, 40) compared to their abundances in controls, while reduced richness of Butyricicoccus was found in patients with inflammatory bowel disease (IBD) (41). Butyricicoccus pullicaecorum is a species of the Butyricicoccus genus that has probiotic potential, demonstrated by its amelioration of colitis in a rat model of inflammatory bowel disease (IBD) and strengthening of the epithelial barrier in a Caco-2 cell model, partially via its butyrate-producing ability (41). On the basis of these findings, we suggest that the higher relative abundances of the genera Gordonibacter, Anaerostipes, Butyricicoccus, and *ventriosum*_group in the feces of vegetarians may positively affect physiological homeostasis. In contrast, the consumption of meat has been associated with the occurrence of CRC, although the detailed mechanism is still under investigation (42). Our results showed that the abundances of the genera Bilophila, Phascolarctobacterium, Tyzzerella, Ruminiclostridium_9, and Negativibacillus in the feces of omnivores tended to be higher than those in vegetarians. Moreover, fecal Actinomyces, Ruminiclostridium_5, and Ruminiclostridium_9 showed a positive correlation with animal protein intakes in omnivores. Previous studies demonstrated that fecal Bilophila (43), Phascolarctobacterium (43, 44), Ruminiclostridium_9 (45), Actinomyces (43), Ruminiclostridium_5 (46), and Ruminiclostridium_9 (45) were relatively enriched in patients with CRC. In addition, the relative abundance of fecal Tyzzerella is associated with lifetime CVD risk (47). Collectively, these data suggest that dietary choice may render an individual prone or resistant to certain diseases by influencing the levels of specific gut bacteria. However, specific criteria for defining healthy versus unhealthy gut flora are still lacking.

In our study, we found that two potential pathobionts, Bilophila and Lachnoclostridium, were sensitive to distinct dietary protein sources. In particular, Bilophila was the only fecal bacterium responsive to dietary fiber, plant-derived protein, and animal-derived protein. In the intestine, Bilophila produces H_2_S and may be involved in the pathogenesis of CRC (14, 43), as well as IBD (2, 48). Notably, the previous studies of mouse models showed that the expansion of Bilophila wadsworthia synergizes with a high-fat diet to aggravate metabolic dysfunction (49) and IBD (48). Therefore, Bilophila has been suggested as an indicator bacterium for diet-related diseases. Additionally, increased fecal Lachnoclostridium has been linked to the formation of visceral fat (50) and progression of diabetic peripheral neuropathy (51). A recent study in Hong Kong identified a Lachnoclostridium gene as a stool-based noninvasive biomarker for early detection of CRC (13). People from Hong Kong, Taiwan, and other East Asian areas share similar eating habits, so further investigations may assess whether the increase in fecal Lachnoclostridium caused by dietary animal protein is associated with the increased risk of CRC in these locations. Our findings also suggest that fecal Lachnoclostridium might be a useful predictor of metabolic-related disease or CRC occurrence that could be modified with dietary intervention.

In conclusion, our study provides evidence that dietary plant and animal proteins oppositely modulate the fecal abundance of Bilophila and Lachnoclostridium in vegetarians and omnivores after bowel cleansing. Understanding the correlations between specific nutrients and specialized fecal bacteria under certain conditions will pave the way for the development of precision nutrition approaches that involve the use of specialized fecal bacteria as biomarkers to guide interventions.

## MATERIALS AND METHODS

### Study population and dietary information.

In total, 46 healthy volunteers, including 23 vegetarians (Veg; 11 lacto-ovo vegetarians, 8 ovo-vegetarians, 2 lacto vegetarians, and 2 vegans) and 23 omnivores (Omn), who were scheduled to receive colonoscopy screening at Taichung Tzu Chi Hospital were recruited to the study (Fig. 1). The inclusion and exclusion criteria are indicated in Fig. 1. The Institutional Review Board (IRB) at Taichung Tzu Chi Hospital (REC107-05) approved the human research protocol, and all participants gave written informed consent before the study.

### Fecal sample collection after bowel cleansing.

This study was conducted from October 2018 to June 2020 in Taichung Tzu Chi Hospital. The demographic information for participants is summarized in Table 1. In total, 46 subjects (20 males, 26 females) with an average age of 55 years (range 29 to 67 years) were evaluated. Bowklean powder (10 mg picosulfate sodium, 3.5 g magnesium oxide, and 12 g citric acid anhydrous) was used for colon cleaning (52–54). Briefly, Bowklean solution was fresh prepared by dissolving one sachet of the powder in 150 mM water and stirring for 5 min. Before the day of colonoscopy, each subject consumed the first Bowklean powder sachet at 5:00 p.m. (17:00), followed by 1,250 mL of clear liquids within 5 h. On the day of the colonoscopy, a second sachet of Bowklean was consumed 5 h prior to the colonoscopy, followed by 750 mL of clear liquids within 2 h. Fecal samples were collected once from each participant between 5 and 7 days after the colonoscopy.

### Dietary assessment. (i) FFQ.

All participants performed a dietary assessment using a semiquantitative food frequency questionnaire (FFQ), adopted from the Nutrition and Health Survey in Taiwan. This survey has been validated in a previous study and can reliably identify major nutrients in the diets of vegetarians and omnivores in Taiwan (55, 56).

### (ii) Image-based dietary assessment.

Sixteen participants (7 vegetarians and 9 omnivores) further participated in the image-based dietary assessment. Participants took photographs of their food with a smart phone before each meal and sent the photographs to a dietitian through the Taiwan social communication app LINE for nutritional evaluation. Participants were encouraged to use a Tzu Chi transparent oval bowl (Fig. S1) provided by the project to facilitate accurate nutritional assessment. Participants had an average diet record of 4 days. Follow-up dietary assessment was performed by two independent dietitians, using the Taiwan Food Ingredient Database (Ministry of Health and Welfare, Taiwan, Republic of China) (57).

### Microbiota analysis.

Methods for fecal sample collection and genomic DNA extraction, 16S rRNA gene amplicon sequencing, and MiSeq-based high-throughput sequencing are described in detail in the supplemental material.

### Statistical analysis. (i) Fecal samples.

The abundances of species at various taxonomic levels were compared among groups using differential-abundance analysis with a zero-inflated Gaussian (ZIG) log-normal model. The analysis was implemented with the “fitFeatureModel” function of the Bioconductor metagenomeSeq package (58). Welch’s *t* test was performed with STAMP software (version 2.1.3) (59). Bacterial richness, alpha diversity, and Firmicutes/Bacteroidetes ratio between groups were examined by Wilcoxon rank sum tests. Permutational multivariate analysis of variance (PERMANOVA) was used to analyze the significance of differences in beta diversity (PCoA). Statistically significant biomarkers were identified from linear discriminant analysis effect size (LEfSe) analysis (60). The LEfSe method involves conducting a nonparametric Kruskal-Wallis test and Wilcoxon rank sum test to determine the bacterial taxa with significantly different relative abundances between groups. Those taxa with an LDA score (log_10_) of >3.0 were represented in a bar plot, but does not mean that they are statistically significantly different. The data analyses were conducted by Biotools Co., Ltd. (New Taipei City, Taiwan).

### (ii) Dietary parameters.

When comparing the nutrient intakes of the two groups (vegetarian and omnivore), the nonparametric Wilcoxon rank sum test was used. Spearman’s correlation was used to examine associations between specific nutrients and fecal bacteria. Statistical tests were performed using GraphPad Prism (version 8).

For all statistical analyses, the significance level α was set as 0.05, and adjusted *P* values (*q* values) were calculated for multiple comparisons using the Benjamini-Hochberg false discovery rate (FDR) correction (61).

### Data availability.

The sequencing data have been submitted to the NCBI SRA database under accession number PRJNA786088.
