# Supplementary material for: Dietary Plant and Animal Protein Sources Oppositely Modulate Fecal Bilophila and Lachnoclostridium in Vegetarians and Omnivores
Source: Microbiol Spectr. 2022 Mar 14;10(2):e02047-21. doi: 10.1128/spectrum.02047-21 (PMC9045121; doi:10.1128/spectrum.02047-21)
Supplement: SUPPLEMENTAL FILE 1 — Supplemental material. Download SPECTRUM02047-21_Supp_1_seq7.pdf, PDF file, 0.8 MB [file spectrum02047-21_supp_1_seq7.pdf]

Supplementary Materials and Methods

Fecal sample collection and genome DNA extraction

Using a stool collection kit (GenoRx Co. Ltd, Taipei, Taiwan), each participant collected a fecal sample at home and sent the sample to the lab in cold storage within 24 h of collection. The samples were then aliquoted and stored at -80°C before use in experiments. Total genomic DNA was extracted from samples using the CTAB/SDS method. DNA concentration and purity were assessed on 1% agarose gels. DNA samples were diluted to 1 ng/µL with sterile water. The DNA extraction protocol was performed by GenoRx Co., Ltd.

16S rRNA gene amplicon sequencing

16S rRNA gene amplicon sequencing was performed by GenoRx Co., Ltd. Briefly, 16S rRNA sequencing libraries were prepared according to the manufacturer's instructions (Illumina, CA, USA). DNA (12.5 ng) was used for PCR amplification of the 16S rRNA gene V3 and V4 regions. The PCR primers contained overhang adapter sequences followed by the full-length primer sequences: Forward: 5′-TCGTCGGCAGCGTCAGATGTGTATAAGAGACAGCCTACGGGNGGCWGCAG; Reverse: 5′-GTCTCGTGGGCTCGGAGATGTGTATAAGAGACAGGACTACHVGGGTATCTAATCC. PCR products were purified with AMPure XP beads (Beckman Coulter, CA, USA). Then, the purified products were subjected to a second PCR with primers from the Nextera XT Index kit (Illumina) to add dual indices and Illumina sequencing adapters. After the second PCR, final libraries (~630 bp) were purified with AMPure XP beads and used in next generation sequencing.

MiSeq-based high throughput sequencing

The concentrations of 16S rRNA sequencing libraries were determined by real-time quantitative PCR with Illumina adapter-specific primers provided in the KAPA library quantification kit (Merck KGaA, Darmstadt, Germany). Libraries were denatured and sequenced using an Illumina Miseq system with reagent V3 for pair-end sequencing (2*250 bp). The Real Time Analysis software (RTA), MiSeq Control software (MCS), and Miseq Report software (MSR) were utilized on the Miseq system, as appropriate. Analyses were performed using FASTQ files generated by MiSeq Report. The method was conducted by GenoRx Co., Ltd.

Data analysis of fecal samples

After demultiplexing, the raw reads were further processed with DADA2 (version 1.10.1) to obtain amplicons with single-nucleotide resolution(1, 2). The DADA2 workflow includes quality filtering, dereplication, learning the dataset-specific error model, ASV inference, and chimera removal. Trimming and filtering were performed with at least 12 perfectly matched bases in each read pair. The DADA2 algorithm resolves exact amplicon sequence variants (ASVs) with single-nucleotide resolution from 16S rRNA gene with a near-zero error rate. For each representative sequence, the RDP classifier (v.2.2) algorithm(3) was used to annotate taxonomy

classification based on the information retrieved from the Silva Database v.132(4, 5). Annotation was performed with an 80% minimum confidence threshold to record an assignment. Sequences with one-time occurrence (singletons) or that were present in only one sample were filtered out. To analyze the sequence similarities among different ASVs, multiple sequence alignment was conducted using PyNAST software (v.1.2)(6) against the core-set dataset in the Silva database.

To normalize the variations in sequence depth across samples, ASV abundance information was rarefied to the minimum sequence depth using the QIIME script (single_rarefaction.py). Subsequent analysis of alpha and beta diversities was both performed on the normalized data. Alpha diversity is indicative of the species complexity within individual samples based on seven different output criteria from the QIIME pipeline, including observed-ASVs, Shannon entropy and Chao index. Observed-ASVs is the number of different species represented in the microbial community. Community richness was assessed by the Chao1 and ACE indices(7). Beta diversity analysis was used to evaluate the differences among samples in terms of species complexity. Principal Coordinate Analysis (PCoA) using Bray–Curtis distance was performed on the distance matrix to acquire principal coordinates for visualization of sophisticated and multidimensional data(8).

References

1. Callahan BJ, McMurdie PJ, Rosen MJ, Han AW, Johnson AJ, Holmes SP. 2016. DADA2: High-resolution sample inference from Illumina amplicon data. Nat Methods 13:581-3.
2. Callahan BJ, McMurdie PJ, Holmes SP. 2017. Exact sequence variants should replace operational taxonomic units in marker-gene data analysis. ISME J 11:2639-2643.
3. Wang Q, Garrity GM, Tiedje JM, Cole JR. 2007. Naive Bayesian classifier for rapid assignment of rRNA sequences into the new bacterial taxonomy. Appl Environ Microbiol 73:5261-7.
4. Quast C, Pruesse E, Yilmaz P, Gerken J, Schweer T, Yarza P, Peplies J, Glockner FO. 2013. The SILVA ribosomal RNA gene database project: improved data processing and web-based tools. Nucleic Acids Res 41:D590-6.
5. Yilmaz P, Parfrey LW, Yarza P, Gerken J, Pruesse E, Quast C, Schweer T, Peplies J, Ludwig W, Glockner FO. 2014. The SILVA and "All-species Living Tree Project (LTP)" taxonomic frameworks. Nucleic Acids Res 42:D643-8.
6. Caporaso JG, Bittinger K, Bushman FD, DeSantis TZ, Andersen GL, Knight R. 2010. PyNAST: a flexible tool for aligning sequences to a template alignment. Bioinformatics 26:266-7.
7. Schloss PD, Westcott SL, Ryabin T, Hall JR, Hartmann M, Hollister EB, Lesniewski RA, Oakley BB, Parks DH, Robinson CJ, Sahl JW, Stres B, Thallinger GG, Van Horn DJ, Weber CF. 2009. Introducing mothur: open-source, platform-independent, community-supported software for describing and comparing microbial communities. Appl Environ Microbiol 75:7537-41.
8. Jiang XT, Peng X, Deng GH, Sheng HF, Wang Y, Zhou HW, Tam NF. 2013. Illumina sequencing of 16S rRNA tag revealed spatial variations of bacterial communities in a mangrove wetland. Microb Ecol 66:96-104.

Supplemental Table 1.
Daily energy and Macronutrient intake of the study participants

	**Vegetarian**	**Omnivore**	***p*-Value**	***q*-Value**
**N**	7	9		
**Age**				
**<65, n (%)**	7 (100)	9 (100)		
**≥65, n (%)**	0 (0)	0 (0)		
**Gender**				
**Male, n (%)**	4 (57.1)	2 (22.2)		
**Female, n (%)**	3 (42.9)	7 (77.8)		
**Energy (kcal)**	1609.7 ± 222.2	1650.6 ± 240.8	0.73	0.94
**Protein (g)**	53.7 ± 11.3	67.8 ± 14.7	0.05	0.22
Plant-derived protein	45.1 ± 12.5	25.3 ± 4.9	<0.01*	**0.02#**
Animal-derived protein	8.5 ± 7.4	42.5 ± 13.5	<0.01*	**0.02#**
**Fat (g)**	61.5 ± 13.9	63.2 ± 18.5	0.84	0.98
**Saturated fat (g)**	14.9 ± 5.6	18.0 ± 5.1	0.26	0.41
**Transfatty acid (mg)**	284.3 ± 116.5	365.7 ± 185.1	0.33	0.43
**Carbohydrate (g)**	219.3 ± 28.5	202.8 ± 30.3	0.38	0.46
**Fiber (g)**	20.4 ± 5.2	13.3 ± 3.9	0.01*	**0.04#**
**Sodium (mg)**	1004.1 ± 513.4	1453.4 ± 716.2	0.18	0.32
**Potasium (mg)**	2013.1 ± 534.9	1971.0 ± 786.5	0.91	1.00
**Cacium (mg)**	594.0 ± 172.6	533.5 ± 365.6	0.69	0.35
**Magnesium (mg)**	291.4 ± 131.2	239.9 ± 99.6	0.39	0.30
**Phosphorus (mg)**	916.1 ± 290.4	893.9 ± 238.8	0.87	0.96
**Cholesterol (mg)**	182.9 ± 102.7	321.0 ± 132.0	0.04*	0.07

Data were presented as mean ± standard deviation.
*$p < 0.05$, #$q < 0.05$ between vegetarians and omnivores.

Supplemental Table 2.
Daily monosaccharide intake of the study participants

	Vegetarian	Omnivore	*p*-Value	*q*-Value
**Glucose (g)**	10.0 ± 4.5	6.1 ± 2.8	0.04*	0.28
**Fructose (g)**	9.2 ± 4.6	5.3 ± 3.1	0.06	0.23
**Maltose (g)**	1.0 ± 0.8	1.2 ± 0.5	0.61	1.00
**Sucrose (g)**	8.9 ± 4.8	9.6 ± 4.1	0.73	0.88
**Lactose (g)**	1.5 ± 3.4	3.5 ± 4.4	0.35	0.73

Data were presented as mean ± standard deviation.
*$p < 0.05$ between vegetarians and omnivores.

Supplemental Table 3.
Daily fatty acid intake of the study participants

	Vegetarian	Omnivore	*p*-Value	*q*-Value
<u>Saturated fats (mg)</u>	14679.1 ± 5770.6	16642.0 ± 5534.9	0.50	0.57
**Butyric acid (C4)**	30.0 ± 62.8	81.1 ± 104.8	0.28	0.53
**Caproic acid (C6)**	28.3 ± 43.8	85.2 ± 86.8	0.14	0.54
**Caprylic acid (C8)**	217.7 ± 442.0	59.1 ± 71.7	0.30	0.78
**Capric acid (C10)**	215.5 ± 442.0	122.2 ± 139.8	0.45	0.83
**Lauric acid (C12)**	1296.0 ± 2651.2	200.6 ± 215.0	0.23	0.55
**Tridecylic acid(C13)**	1.9 ± 2.3	3.2 ± 3.7	0.33	0.59
**Myristic acid (C14)**	724.9 ± 917.7	740.9 ± 469.9	0.97	0.54
**Pentadecanoic acid (C15)**	2.0 ± 2.6	5.8 ± 3.7	0.04*	0.07
**Palmitic acid (C16)**	8629.4 ± 1630.9	10888.5 ± 3557.0	0.14	0.41
**Margaric acid (C17)**	59.0 ± 23.5	106.1 ± 36.7	0.01*	0.09
**Stearic acid (C18)**	2824.5 ± 853.5	3854.4 ± 1224.8	0.11	0.26
**Heptadecanoic acid (C19)**	5.0 ± 13.1	3.9 ± 4.2	0.83	0.08
**Arachidic acid (C20)**	203.7 ±63.5	192.6 ± 54.7	0.70	0.89
**Behenic acid (C22)**	217.0 ± 55.7	175.8 ± 38.7	0.13	0.38
**Lignoceric acid (C24)**	106.4 ± 39.2	69.0 ± 14.8	0.02*	0.08
<u>Monounsaturated fats (mg)</u>	17365.7 ± 3556.3	19040.5 ± 6191.6	0.54	0.86
**Myristol**	6.5 ± 9.3	18.8 ± 20.4	0.16	0.24
**Palmitoyl 16:1 (Palmitic acid)**	293.9 ± 79.7	738.4 ± 323.9	<0.01*	0.05#
**Oleic acid**	16898.1 ± 3512.3	17922.3 ± 5716.8	0.68	1.00
**Gadoleic Acid 20:1**	154.0 ± 36.1	310.6 ± 177.4	0.04*	0.07
**Erucic acid**	13.1 ± 5.0	50.4 ± 44.9	0.05*	0.07
<u>Polyunsaturated fats (mg)</u>	28280.3 ± 7242.2	24857.7 ± 9923.8	0.46	0.51
**Linoleic acid (LA) (g)**	25.1 ± 6.6	21.6 ± 8.5	0.38	0.49
**α-Linolenic acid (g)**	3.0 ± 0.7	2.5 ± 1.1	0.33	0.51
**Stearidonic acid (SDA)**	0.4 ± 0.5	19.4 ± 28.7	0.10	0.10
**Arachidonic acid (ETA)**	70.2 ± 34.5	174.9 ± 90.6	0.01*	0.07
**Eicosapentaenoic (EPA)**	10.0 ± 12.4	126.4 ± 151.0	0.06	0.07
**Clupanodonic acid (DPA)**	7.1 ± 4.4	95.9 ± 85.1	0.02*	0.02#
**Docosahexaenoic Acid (DHA)**	39.3 ± 20.0	290.3 ± 276.4	0.03*	0.11

Data were presented as mean ± standard deviation.
*$p < 0.05$, #$q < 0.05$ between vegetarians and omnivores.

Supplemental Table 4.
Daily amino acid intake of the study participants

Amino acid (mg)	Vegetarian	Omnivore	*p*-Value	*q*-Value
**Essential amino acids**				
**Threonine**	1773.7 ± 383.5	2430.7 ± 735.7	0.05	0.18
**Valine**	2287.5 ± 514.9	3026.8 ± 800.5	0.09	0.28
**Methionine**	772.9 ± 171.4	1367.1 ± 463.3	0.01*	0.20
**Isoleucine**	2075.3 ± 449.4	2644.5 ± 728.7	0.09	0.30
**Leucine**	3900.5 ± 877.0	4942.3 ± 1348.1	0.10	0.28
**Phenylalanine**	2506.3 ± 570.0	2850.7 ± 712.3	0.31	0.48
**Lysine**	2408.5 ± 553.0	3875.1 ± 1419.6	0.02*	0.27
**Histidine**	1267.1 ± 277.1	1817.3 ± 512.3	0.02*	0.20
**Tryptophan**	437.0 ± 118.5	658.3 ± 209.0	0.03*	0.16
**Non-essential amino acids**				
**Aspartic acid**	4398.9 ± 1046.9	5408.1 ± 1720.3	0.19	0.34
**Serine**	2503.6 ± 538.9	2795.4 ± 765.7	0.41	0.56
**Glutamate**	10751.1 ± 2786.2	11623.8 ± 2375.7	0.51	0.53
**Proline**	3424.0 ± 912.7	3655.7 ± 537.8	0.54	0.44
**Glycine**	1975.5 ± 494.8	2673.1 ± 712.9	0.05*	0.13
**Alanine**	2216.5 ± 462.5	3152.3 ± 937.7	0.03*	0.16
**Cysteine**	1529.2 ± 322.1	2111.1± 467.8	0.01*	0.24
**Tyrosine**	1850.2 ± 443.1	2303.1 ± 605.3	0.12	0.25
**Arginine**	3179.5 ± 882.5	3822.7 ± 1059.2	0.22	0.45

Data were presented as mean ± standard deviation.
*$p < 0.05$ between vegetarians and omnivores.

Supplemental Table 5.
Daily micronutrient intake of the study participants

	Vegetarian	Omnivore	*p*-Value	*q*-Value
**Iron (mg)**	11.8 ± 2.9	13.1 ± 7.8	0.69	0.92
**Zinc (mg)**	7.7 ± 1.8	9.5 ± 2.3	0.12	0.39
**Copper (mg)**	0.1 ± 0.1	0.1 ± 0.0	0.20	0.60
**Manganese (mg)**	0.3 ± 0.5	0.0 ± 0.0	0.19	0.73
**Vitamin A (IU)**	10261.0 ± 3527.7	6628.4 ± 3869.9	0.07	0.34
**Retinol (RE) (μg)**	1103.7 ± 352.1	764.2 ± 374.6	0.09	0.36
**Alpha carotene (μg)**	666.0 ± 616.2	268.8 ± 163.6	0.08	0.43
**Beta carotene (μg)**	5591.5 ± 1968.8	3558.6 ± 2285.8	0.08	0.39
**Vitamin E (mg)**	50.9 ± 34.0	33.2 ± 13.3	0.17	0.39
**α-tocopherol (mg)**	21.9 ± 27.3	9.3 ± 6.4	0.20	0.57
**β-tocopherol (mg)**	1.4 ± 0.6	1.0 ± 0.5	0.21	0.40
**γ-tocopherol (mg)**	22.0 ± 6.3	18.2 ± 7.5	0.30	0.41
**δ-tocopherol (mg)**	5.7 ± 1.7	4.6 ± 1.8	0.24	0.36
**Thiamin (mg)**	0.8 ± 0.3	1.0 ± 0.4	0.52	0.38
**Riboflavin (mg)**	0.9 ± 0.5	1.0 ± 0.2	0.62	0.32
**Niacin (mg NE)**	15.6 ± 4.2	20.4 ± 3.9	0.02*	0.21
**Pyridoxine (mg)**	1.2 ± 0.3	1.3 ± 0.2	0.36	0.37
**Cobalamin (μg)**	1.20± 1.1	4.1 ± 2.8	0.02*	0.12
**Folate (μg)**	304.2 ± 66.4	229.2 ± 56.7	0.03*	0.30
**Ascorbic acid (mg)**	116.8 ± 23.6	113.2 ± 59.9	0.88	0.74

Supplemental Table 6.
Linear discriminant analysis (LDA) effect size (LEfSe) analysis

Taxonomy	Group	LDA score	*p*-value	*q*-value*
p__Firmicutes.c__Bacilli.o__Lactobacillales.f__Lactobacillaceae.g__Lactobacillus	Veg	3.82	0.04	ns
p__Firmicutes.c__Bacilli.o__Lactobacillales.f__Lactobacillaceae	Veg	3.82	0.04	ns
p__Fusobacteria.c__Fusobacteriia.o__Fusobacteriales.f__Fusobacteriaceae	Veg	3.78	0.02	ns
p__Firmicutes.c__Bacilli.o__Lactobacillales.f__Carnobacteriaceae	Veg	3.78	0.04	ns
p__Fusobacteria.c__Fusobacteriia.o__Fusobacteriales	Veg	3.77	0.03	ns
p__Fusobacteria.c__Fusobacteriia	Veg	3.75	0.03	ns
p__Fusobacteria	Veg	3.74	0.03	ns
p__Fusobacteria.c__Fusobacteriia.o__Fusobacteriales.f__Fusobacteriaceae.g__Fusobacterium	Veg	3.74	0.03	ns
p__Firmicutes.c__Negativicutes.o__Selenomonadales.f__Veillonellaceae.g__Megasphaera	Veg	3.67	0.04	ns
p__Firmicutes.c__Clostridia.o__Clostridiales.f__Lachnospiraceae.g___Eubacterium__ventriosum_group	Veg	3.52	0.02	ns
p__Proteobacteria.c__Gammaproteobacteria.o__Enterobacteriales.f__Enterobacteriaceae.g__Klebsiella	Veg	3.49	0.04	ns
p__Firmicutes.c__Clostridia.o__Clostridiales.f__Lachnospiraceae.g__Roseburia.s__unidentified	Veg	3.34	0.02	ns
p__Firmicutes.c__Bacilli.o__Lactobacillales.f__Lactobacillaceae.g__Lactobacillus.s__Lactobacillus_gasseri	Veg	3.26	0.04	ns
p__Firmicutes.c__Clostridia.o__Clostridiales.f__Ruminococcaceae.g__Ruminiclostridium_5	Omn	3.13	0.00	ns
k__Archaea.p__Nanoarchaeaeota.c__Nanohaloarchaeia	Omn	3.48	0.04	ns
k__Archaea.p__Nanoarchaeaeota.c__Nanohaloarchaeia.o__Deep_Sea_Euryarchaeotic_GroupDSEG	Omn	3.54	0.01	ns
p__Firmicutes.c__Negativicutes.o__Selenomonadales.f__Acidaminococcaceae	Omn	3.94	0.01	ns
p__Firmicutes.c__Negativicutes.o__Selenomonadales.f__Acidaminococcaceae.g__Phascolarctobacterium	Omn	3.96	0.03	ns
p__Bacteroidetes.c__Bacteroidia.o__Bacteroidales.f__Prevotellaceae.g__Prevotella_9	Omn	3.98	0.03	ns
p__Bacteroidetes.c__Bacteroidia.o__Bacteroidales.f__Prevotellaceae	Omn	4.10	0.03	ns
p__Firmicutes.c__Negativicutes	Omn	4.34	0.04	ns
p__Firmicutes.c__Negativicutes.o__Selenomonadales	Omn	4.34	0.04	ns

The threshold on the logarithmic LDA score for discriminative features was set to 3.0.
Abbreviation: "p", phylum; "c", class; "o", order; "f", family; "g", genus; "veg", vegetarians; "omn" omnivores.
$p < 0.05$ are considered statistically significant.
*ns: non-significant

Supplemental Table 7

	Phylum	p_Actinobacteria			Class	c_Woesearchaeia			c_Coriobacteriia		
		Spearman Correlation	*p* value	*q* value		Spearman Correlation	*p* value	*q* value	Spearman Correlation	p value	*q* value
**Tryptophan**		0.47	0.07	0.47		-0.65	***0.01***	0.27	0.45	0.08	0.26
**Histidine**		0.38	0.15	0.34		-0.62	***0.01***	0.17	0.40	0.13	0.28
**Lysine**		0.39	0.13	0.35		-0.61	***0.01***	0.16	0.39	0.14	0.28
**Methionine**		0.50	***0.05***	0.51		-0.64	***0.01***	0.17	0.52	***0.04***	0.20
**Lignoceric acid**		0.02	0.93	0.93		0.13	0.64	0.85	-0.03	0.92	0.98
**Docosahexaenoic Acid**		0.37	0.15	0.32		-0.37	0.16	0.31	0.48	0.06	0.25
**Clupanodonic acid**		0.28	0.29	0.48		-0.34	0.19	0.35	0.43	0.10	0.27
**Arachidonic acid**		0.45	0.08	0.42		-0.40	0.12	0.29	0.55	***0.03***	0.20
**Erucic acid**		0.10	0.72	0.80		-0.12	0.66	0.84	0.16	0.56	0.81
**Gadoleic acid**		0.26	0.32	0.48		-0.43	0.10	0.25	0.39	0.14	0.28
**Palmitic acid**		0.40	0.12	0.43		-0.55	***0.03***	0.17	0.46	0.07	0.24
**Margaric acid**		0.32	0.22	0.39		-0.56	***0.02***	0.23	0.40	0.12	0.28
**Pentadecanoic acid**		0.23	0.39	0.55		-0.44	0.09	0.27	0.31	0.24	0.41
**Cholesterol**		0.53	***0.04***	0.76		-0.39	0.13	0.28	0.55	***0.03***	0.22
**Cobalamin**		0.39	0.13	0.39		-0.47	0.06	0.23	0.48	0.06	0.25
**Niacin**		0.41	0.11	0.48		-0.67	***<0.01***	0.40	0.43	0.10	0.26
**Animal-derived Protein**		0.33	0.21	0.40		-0.48	0.06	0.26	0.43	0.09	0.27
**Folic acid**		0.11	0.69	0.85		-0.07	0.79	0.92	-0.03	0.92	0.97
**Glucose**		-0.17	0.53	0.69		0.18	0.51	0.74	-0.21	0.43	0.68
**Plant-derived Protein**		0.11	0.69	0.80		0.10	0.71	0.86	-0.07	0.81	0.92
**Fiber**		0.07	0.80	0.84		0.07	0.80	0.92	-0.14	0.62	0.86

Supplemental Table 7 (continued)

	c_Bacteroidia			c_Deltaproteobacteria			Order	o_Actinomycetales		
	Spearman Correlation	*p* value	*q* value	Spearman Correlation	*p* value	*q* value		Spearman Correlation	*p* value	*q* value
**Tryptophan**	-0.01	0.97	0.98	0.20	0.46	0.72		-0.08	0.78	1.00
**Histidine**	0.13	0.64	0.84	0.32	0.23	0.41		-0.21	0.44	1.00
**Lysine**	-0.03	0.92	0.96	0.27	0.31	0.52		-0.14	0.60	1.00
**Methionine**	0.04	0.88	0.99	0.35	0.18	0.34		-0.21	0.44	1.00
**Lignoceric acid**	0.04	0.88	0.97	-0.40	0.13	0.28		-0.05	0.85	0.98
**Docosahexaenoic Acid**	-0.11	0.69	0.85	0.52	***0.04***	0.19		-0.24	0.38	1.00
**Clupanodonic acid**	-0.14	0.61	0.87	0.47	0.06	0.24		-0.40	0.12	0.89
**Arachidonic acid**	-0.13	0.63	0.85	0.46	0.07	0.24		-0.26	0.34	1.00
**Erucic acid**	0.20	0.46	0.71	0.42	0.11	0.26		-0.36	0.17	0.87
**Gadoleic acid**	-0.12	0.66	0.83	0.27	0.31	0.52		-0.44	0.09	0.90
**Palmitic acid**	0.08	0.76	0.91	0.47	0.07	0.24		-0.17	0.54	1.00
**Margaric acid**	0.14	0.62	0.85	0.57	***0.02***	0.22		-0.22	0.40	1.00
**Pentadecanoic acid**	0.50	***0.05***	0.23	0.55	***0.03***	0.18		-0.07	0.79	1.00
**Cholesterol**	0.02	0.93	0.95	0.43	0.10	0.25		-0.17	0.54	1.00
**Cobalamin**	-0.03	0.91	1.00	0.59	***0.02***	0.18		-0.23	0.38	1.00
**Niacin**	-0.03	0.92	0.99	0.22	0.42	0.67		-0.07	0.79	1.00
**Animal-derived Protein**	0.19	0.47	0.71	0.55	***0.03***	0.19		-0.47	0.06	0.82
**Folic acid**	-0.08	0.77	0.91	-0.36	0.18	0.33		0.56	***0.02***	0.97
**Glucose**	0.12	0.65	0.84	-0.35	0.18	0.33		0.43	0.10	0.84
**Plant-derived Protein**	-0.40	0.12	0.29	-0.53	***0.03***	0.19		0.50	***0.05***	0.82
**Fiber**	-0.01	0.97	0.97	-0.65	***0.01***	0.18		0.29	0.27	1.00

Supplemental Table 7 (continued)

	o_Micrococcales			o_Bacteroidales			o_Bacillales		
	Spearman Correlation	*p* value	*q* value	Spearman Correlation	*p* value	*q* value	Spearman Correlation	*p* value	*q* value
**Tryptophan**	-0.17	0.53	1.00	0.21	0.42	1.00	-0.13	0.63	1.00
**Histidine**	-0.12	0.66	1.00	0.20	0.45	1.00	-0.12	0.66	1.00
**Lysine**	-0.14	0.60	1.00	0.14	0.60	1.00	-0.14	0.60	1.00
**Methionine**	-0.40	0.12	0.87	0.14	0.59	1.00	-0.18	0.51	1.00
**Lignoceric acid**	-0.01	0.97	0.99	0.48	0.06	0.87	0.08	0.75	1.00
**Docosahexaenoic Acid**	-0.55	***0.03***	0.93	-0.07	0.80	0.98	-0.33	0.21	0.97
**Clupanodonic acid**	-0.44	0.09	0.85	-0.06	0.82	0.98	-0.38	0.15	0.86
**Arachidonic acid**	-0.58	***0.02***	1.00	-0.08	0.76	1.00	-0.28	0.29	1.00
**Erucic acid**	0.03	0.91	0.99	0.13	0.63	1.00	-0.59	***0.02***	1.00
**Gadoleic acid**	-0.42	0.11	0.88	0.28	0.29	1.00	-0.29	0.27	1.00
**Palmitic acid**	-0.11	0.69	1.00	-0.14	0.59	1.00	-0.20	0.47	1.00
**Margaric acid**	-0.10	0.72	1.00	0.08	0.76	1.00	-0.23	0.39	1.00
**Pentadecanoic acid**	0.08	0.78	1.00	0.05	0.86	0.98	-0.18	0.51	1.00
**Cholesterol**	-0.53	***0.03***	0.94	-0.10	0.72	1.00	-0.25	0.35	1.00
**Cobalamin**	-0.50	***0.05***	0.90	-0.19	0.49	1.00	-0.30	0.26	1.00
**Niacin**	0.02	0.94	0.98	-0.08	0.76	1.00	***<0.01***	0.99	1.00
**Animal-derived Protein**	-0.40	0.12	0.84	-0.12	0.66	1.00	-0.22	0.41	1.00
**Folic acid**	0.53	***0.04***	0.84	0.15	0.58	1.00	0.27	0.32	1.00
**Glucose**	0.19	0.49	1.00	-0.04	0.88	0.99	0.66	***0.01***	0.92
**Plant-derived Protein**	0.14	0.60	1.00	0.40	0.12	0.81	0.16	0.55	1.00
**Fiber**	0.38	0.15	0.84	0.30	0.26	1.00	0.43	0.10	0.80

Supplemental Table 7 (continued)

	o_Lactobacillales			o_Rickettsiales			o_Desulfovibrionales		
	**Spearman Correlation**	***p* value**	***q* value**	**Spearman Correlation**	***p* value**	***q* value**	**Spearman Correlation**	***p* value**	***q* value**
**Tryptophan**	0.07	0.79	1.00	0.04	0.88	0.98	-0.06	0.82	0.99
**Histidine**	-0.12	0.66	1.00	0.15	0.58	1.00	-0.17	0.53	1.00
**Lysine**	-0.05	0.86	0.99	0.09	0.75	1.00	-0.10	0.73	1.00
**Methionine**	0.03	0.92	0.98	0.02	0.94	0.97	-0.19	0.48	1.00
**Lignoceric acid**	-0.46	0.08	0.85	0.03	0.91	0.98	0.35	0.19	0.91
**Docosahexaenoic Acid**	0.18	0.50	1.00	-0.10	0.72	1.00	-0.27	0.32	1.00
**Clupanodonic acid**	0.28	0.30	1.00	-0.12	0.66	1.00	-0.50	***0.05***	0.98
**Arachidonic acid**	0.14	0.62	1.00	-0.04	0.88	0.99	-0.28	0.30	1.00
**Erucic acid**	-0.09	0.74	1.00	0.39	0.14	0.82	-0.19	0.48	1.00
**Gadoleic acid**	-0.06	0.82	0.98	0.07	0.80	0.99	-0.26	0.32	1.00
**Palmitic acid**	0.24	0.37	1.00	0.09	0.74	1.00	-0.33	0.22	0.98
**Margaric acid**	0.07	0.80	0.99	-0.12	0.66	1.00	-0.44	0.09	0.84
**Pentadecanoic acid**	0.04	0.89	0.98	0.06	0.83	0.98	-0.47	0.06	0.77
**Cholesterol**	0.11	0.67	1.00	-0.02	0.93	0.98	-0.19	0.48	1.00
**Cobalamin**	0.10	0.70	1.00	-0.07	0.80	0.99	-0.36	0.18	0.88
**Niacin**	0.12	0.66	1.00	0.13	0.64	1.00	-0.12	0.67	1.00
**Animal-derived Protein**	-0.03	0.91	0.99	0.15	0.58	1.00	-0.40	0.13	0.78
**Folic acid**	-0.05	0.86	0.99	***<0.01***	0.99	0.99	0.24	0.37	1.00
**Glucose**	0.03	0.93	0.98	-0.03	0.91	0.98	0.36	0.17	0.87
**Plant-derived Protein**	0.06	0.82	0.98	-0.38	0.15	0.81	0.48	0.06	0.92
**Fiber**	-0.17	0.52	1.00	-0.01	0.96	0.98	0.41	0.11	0.87

Supplemental Table 7 (continued)

	o_Pasteurellales			Family	f_Actinomycetaceae			f_Coriobacteriaceae		
	Spearman Correlation	*p* value	*q* value		Spearman Correlation	*p* value	*q* value	Spearman Correlation	p value	Adj. p value
**Tryptophan**	0.11	0.69	1.00		0.67	***<0.01***	0.19	0.34	0.20	0.59
**Histidine**	0.10	0.72	1.00		0.66	***0.01***	0.14	0.26	0.33	0.68
**Lysine**	0.16	0.55	1.00		0.66	***0.01***	0.13	0.26	0.33	0.68
**Methionine**	0.01	0.98	0.99		0.80	***<0.01***	***0.05***	0.40	0.12	0.52
**Lignoceric acid**	-0.13	0.64	1.00		0.03	0.91	0.99	-0.04	0.88	0.99
**Docosahexaenoic Acid**	0.23	0.39	1.00		0.65	***0.01***	0.13	0.41	0.12	0.51
**Clupanodonic acid**	0.23	0.39	1.00		0.62	***0.01***	0.17	0.29	0.28	0.67
**Arachidonic acid**	0.16	0.56	1.00		0.68	***<0.01***	0.27	0.43	0.09	0.48
**Erucic acid**	0.33	0.22	0.97		0.26	0.33	0.68	0.10	0.72	0.90
**Gadoleic acid**	0.29	0.28	1.00		0.67	***<0.01***	0.19	0.22	0.41	0.74
**Palmitic acid**	0.17	0.54	1.00		0.53	***0.04***	0.26	0.41	0.11	0.51
**Margaric acid**	0.20	0.47	1.00		0.60	***0.01***	0.18	0.38	0.15	0.54
**Pentadecanoic acid**	-0.21	0.43	1.00		0.51	***0.04***	0.28	0.27	0.30	0.68
**Cholesterol**	-0.06	0.84	0.97		0.66	***0.01***	0.15	0.50	***0.05***	0.30
**Cobalamin**	-0.03	0.91	1.00		0.76	***<0.01***	0.07	0.28	0.30	0.68
**Niacin**	0.12	0.66	1.00		0.53	***0.04***	0.27	0.29	0.27	0.67
**Animal-derived Protein**	-0.02	0.93	0.97		0.68	***<0.01***	0.23	0.24	0.36	0.71
**Folic acid**	-0.07	0.79	1.00		-0.18	0.51	0.78	0.10	0.70	0.89
**Glucose**	-0.24	0.38	1.00		-0.43	0.10	0.48	0.01	0.97	1.00
**Plant-derived Protein**	0.16	0.56	1.00		-0.19	0.47	0.76	0.13	0.64	0.86
**Fiber**	-0.17	0.53	1.00		-0.37	0.16	0.56	0.03	0.91	0.99

Supplemental Table 7 (continued)

	f_Eggerthellaceae			f_Bacteroidaceae			f_Tannerellaceae		
	**Spearman Correlation**	***p* value**	***q* value**	**Spearman Correlation**	***p* value**	***q* value**	**Spearman Correlation**	***p* value**	***q* value**
**Tryptophan**	0.40	0.12	0.53	-0.04	0.89	0.99	***<0.01***	0.99	1.00
**Histidine**	0.46	0.07	0.41	0.06	0.82	0.94	0.06	0.82	0.95
**Lysine**	0.40	0.13	0.51	-0.08	0.78	0.93	-0.01	0.97	1.00
**Methionine**	0.57	***0.02***	0.25	-0.04	0.88	0.98	0.09	0.74	0.91
**Lignoceric acid**	0.10	0.71	0.90	0.13	0.64	0.87	-0.07	0.79	0.93
**Docosahexaenoic Acid**	0.42	0.11	0.51	-0.19	0.47	0.76	0.04	0.88	0.99
**Clupanodonic acid**	0.36	0.17	0.57	-0.23	0.40	0.75	0.02	0.94	1.00
**Arachidonic acid**	0.52	***0.04***	0.28	-0.23	0.40	0.74	-0.02	0.95	1.00
**Erucic acid**	-0.01	0.97	1.00	0.18	0.51	0.78	0.07	0.79	0.93
**Gadoleic acid**	0.39	0.13	0.52	-0.21	0.44	0.76	-0.12	0.66	0.88
**Palmitic acid**	0.33	0.21	0.59	-0.04	0.89	0.98	0.27	0.31	0.67
**Margaric acid**	0.44	0.09	0.48	0.01	0.97	1.00	0.23	0.39	0.75
**Pentadecanoic acid**	0.37	0.15	0.55	0.40	0.13	0.52	0.54	***0.03***	0.26
**Cholesterol**	0.54	***0.03***	0.27	-0.07	0.80	0.93	0.19	0.49	0.78
**Cobalamin**	0.56	***0.02***	0.24	-0.09	0.75	0.92	-0.02	0.94	1.00
**Niacin**	0.35	0.19	0.59	-0.09	0.73	0.91	0.07	0.79	0.94
**Animal-derived Protein**	0.55	***0.03***	0.26	0.09	0.75	0.92	0.18	0.50	0.78
**Folic acid**	-0.11	0.68	0.89	-0.08	0.77	0.93	0.03	0.90	0.99
**Glucose**	-0.16	0.56	0.82	0.08	0.76	0.92	0.34	0.20	0.59
**Plant-derived Protein**	-0.18	0.52	0.78	-0.28	0.29	0.68	-0.31	0.25	0.63
**Fiber**	-0.10	0.70	0.90	0.02	0.93	1.00	***<0.01***	0.99	0.99

Supplemental Table 7 (continued)

	f_Lactobacillaceae			f_Clostridiaceae_1			f_Lachnospiraceae		
	**Spearman Correlation**	***p* value**	***q* value**	**Spearman Correlation**	***p* value**	***q* value**	**Spearman Correlation**	***p* value**	***q* value**
**Tryptophan**	0.29	0.27	0.67	0.20	0.47	0.76	0.33	0.21	0.59
**Histidine**	0.28	0.29	0.67	0.18	0.52	0.77	0.29	0.27	0.66
**Lysine**	0.29	0.28	0.67	0.21	0.44	0.76	0.31	0.24	0.63
**Methionine**	0.37	0.16	0.56	0.34	0.20	0.59	0.23	0.40	0.74
**Lignoceric acid**	0.15	0.59	0.85	-0.05	0.86	0.98	0.09	0.74	0.92
**Docosahexaenoic Acid**	0.26	0.34	0.68	0.23	0.40	0.73	0.03	0.92	0.99
**Clupanodonic acid**	0.11	0.69	0.90	0.15	0.58	0.84	***<0.01***	1.00	1.00
**Arachidonic acid**	0.34	0.20	0.60	0.32	0.23	0.62	0.11	0.67	0.89
**Erucic acid**	-0.18	0.50	0.78	-0.05	0.87	0.98	0.02	0.95	0.99
**Gadoleic acid**	0.20	0.45	0.76	0.14	0.60	0.85	0.11	0.67	0.88
**Palmitic acid**	0.11	0.69	0.90	0.25	0.35	0.70	0.20	0.45	0.75
**Margaric acid**	0.23	0.38	0.74	0.18	0.50	0.78	0.02	0.94	1.00
**Pentadecanoic acid**	0.21	0.43	0.76	0.27	0.30	0.67	-0.01	0.97	0.99
**Cholesterol**	0.35	0.19	0.59	0.42	0.11	0.50	0.09	0.74	0.91
**Cobalamin**	0.38	0.15	0.55	0.37	0.16	0.55	0.13	0.64	0.87
**Niacin**	0.20	0.45	0.75	0.28	0.30	0.68	0.46	0.07	0.40
**Animal-derived Protein**	0.13	0.64	0.87	0.24	0.37	0.73	0.16	0.56	0.82
**Folic acid**	0.33	0.21	0.59	0.21	0.44	0.76	0.16	0.56	0.81
**Glucose**	0.07	0.79	0.93	-0.07	0.80	0.93	-0.03	0.91	0.99
**Plant-derived Protein**	0.26	0.33	0.69	-0.14	0.60	0.86	0.01	0.98	1.00
**Fiber**	0.03	0.90	0.99	-0.06	0.82	0.94	0.14	0.61	0.85

Supplemental Table 7 (continued)

	f_Peptostreptococcaceae			f_Acidaminococcaceae			f_Desulfovibrionaceae		
	**Spearman Correlation**	***p* value**	***q* value**	**Spearman Correlation**	***p* value**	***q* value**	**Spearman Correlation**	***p* value**	***q* value**
**Tryptophan**	0.61	***0.01***	0.18	0.13	0.62	0.86	0.20	0.46	0.76
**Histidine**	0.58	***0.02***	0.23	0.27	0.31	0.68	0.32	0.23	0.62
**Lysine**	0.60	***0.01***	0.19	0.16	0.56	0.82	0.27	0.31	0.68
**Methionine**	0.53	***0.03***	0.26	0.20	0.45	0.76	0.35	0.18	0.60
**Lignoceric acid**	0.20	0.46	0.75	-0.23	0.40	0.76	-0.40	0.13	0.53
**Docosahexaenoic Acid**	0.25	0.35	0.70	0.18	0.51	0.77	0.52	***0.04***	0.27
**Clupanodonic acid**	0.14	0.60	0.85	0.23	0.40	0.75	0.47	0.06	0.37
**Arachidonic acid**	0.31	0.24	0.63	0.13	0.62	0.86	0.46	0.07	0.39
**Erucic acid**	0.22	0.42	0.74	0.50	***0.05***	0.31	0.42	0.11	0.51
**Gadoleic acid**	0.39	0.14	0.52	0.12	0.66	0.88	0.27	0.31	0.68
**Palmitic acid**	0.40	0.12	0.53	0.39	0.14	0.51	0.47	0.07	0.40
**Margaric acid**	0.29	0.27	0.67	0.33	0.22	0.60	0.57	***0.02***	0.24
**Pentadecanoic acid**	0.28	0.30	0.68	0.53	***0.03***	0.28	0.55	***0.03***	0.27
**Cholesterol**	0.32	0.22	0.61	0.21	0.43	0.76	0.43	0.10	0.48
**Cobalamin**	0.30	0.26	0.67	0.22	0.40	0.74	0.59	***0.02***	0.20
**Niacin**	0.63	***0.01***	0.16	0.19	0.49	0.78	0.22	0.42	0.75
**Animal-derived Protein**	0.27	0.31	0.67	0.48	0.06	0.35	0.55	***0.03***	0.27
**Folic acid**	0.39	0.14	0.52	-0.34	0.19	0.59	-0.36	0.18	0.58
**Glucose**	-0.01	0.96	1.00	-0.17	0.54	0.80	-0.35	0.18	0.59
**Plant-derived Protein**	0.14	0.61	0.85	-0.66	***0.01***	0.17	-0.53	***0.03***	0.27
**Fiber**	0.10	0.72	0.90	-0.34	0.20	0.59	-0.65	***0.01***	0.14

Supplemental Table 7 (continued)

Genus	g_Actinomyces			g_Collinsella			g_Raoultibacter		
	Spearman Correlation	*p* value	*q* value	Spearman Correlation	*p* value	*q* value	Spearman Correlation	*p* value	*q* value
**Tryptophan**	0.64	***0.01***	0.33	0.34	0.20	0.63	0.46	0.07	0.49
**Histidine**	0.65	***0.01***	0.34	0.26	0.33	0.74	0.51	***0.04***	0.45
**Lysine**	0.64	***0.01***	0.30	0.26	0.33	0.74	0.57	***0.02***	0.37
**Methionine**	0.78	***<0.01***	0.21	0.40	0.12	0.57	0.50	***0.05***	0.46
**Lignoceric acid**	0.06	0.83	0.98	-0.04	0.88	0.99	-0.01	0.97	0.99
**Docosahexaenoic Acid**	0.61	***0.01***	0.29	0.41	0.12	0.56	0.33	0.22	0.65
**Clupanodonic acid**	0.58	***0.02***	0.36	0.29	0.28	0.70	0.22	0.42	0.83
**Arachidonic acid**	0.65	***0.01***	0.41	0.43	0.09	0.53	0.39	0.13	0.56
**Erucic acid**	0.21	0.43	0.83	0.10	0.72	0.96	0.38	0.15	0.57
**Gadoleic acid**	0.65	***0.01***	0.41	0.22	0.41	0.82	0.32	0.22	0.65
**Palmitic acid**	0.49	0.05	0.46	0.41	0.11	0.55	0.46	0.07	0.50
**Margaric acid**	0.60	***0.01***	0.34	0.38	0.15	0.57	0.43	0.10	0.52
**Pentadecanoic acid**	0.53	***0.03***	0.44	0.27	0.30	0.72	0.32	0.23	0.66
**Cholesterol**	0.64	***0.01***	0.31	0.50	***0.05***	0.45	0.47	0.06	0.48
**Cobalamin**	0.74	***<0.01***	0.17	0.28	0.30	0.71	0.46	0.08	0.50
**Niacin**	0.49	0.05	0.45	0.29	0.27	0.67	0.50	0.05	0.45
**Animal-derived Protein**	0.66	***<0.01***	0.45	0.24	0.36	0.78	0.40	0.13	0.56
**Folic acid**	-0.16	0.56	0.90	0.10	0.70	0.96	0.31	0.25	0.67
**Glucose**	-0.41	0.12	0.56	0.01	0.97	0.99	-0.45	0.08	0.51
**Plant-derived Protein**	-0.18	0.49	0.85	0.13	0.64	0.94	-0.07	0.78	0.98
**Fiber**	-0.34	0.20	0.63	0.03	0.91	0.99	-0.04	0.89	0.98

Supplemental Table 7 (continued)

	g_Eggerthella	g_Bacteroides	g_Parabacteroides

	Spearman Correlation	*p* value	*q* value	Spearman Correlation	*p* value	*q* value	Spearman Correlation	*p* value	*q* value
**Tryptophan**	0.27	0.31	0.73	-0.04	0.89	0.98	***<0.01***	0.99	1.00
**Histidine**	0.27	0.31	0.72	0.06	0.82	0.98	0.06	0.82	0.98
**Lysine**	0.24	0.38	0.79	-0.08	0.78	0.98	-0.01	0.97	0.99
**Methionine**	0.19	0.48	0.85	-0.04	0.88	0.98	0.09	0.74	0.97
**Lignoceric acid**	0.40	0.13	0.57	0.13	0.64	0.93	-0.07	0.79	0.97
**Docosahexaenoic Acid**	-0.05	0.85	0.98	-0.19	0.47	0.85	0.04	0.88	0.99
**Clupanodonic acid**	-0.16	0.55	0.90	-0.23	0.40	0.81	0.02	0.94	0.99
**Arachidonic acid**	0.03	0.92	0.98	-0.23	0.40	0.81	-0.02	0.95	0.99
**Erucic acid**	-0.16	0.57	0.90	0.18	0.51	0.86	0.07	0.79	0.97
**Gadoleic acid**	0.07	0.80	0.97	-0.21	0.44	0.83	-0.12	0.66	0.94
**Palmitic acid**	-0.12	0.66	0.94	-0.04	0.89	0.98	0.27	0.31	0.73
**Margaric acid**	-0.01	0.97	0.99	0.01	0.97	0.99	0.23	0.39	0.81
**Pentadecanoic acid**	0.04	0.89	0.98	0.40	0.13	0.56	0.54	***0.03***	0.44
**Cholesterol**	0.01	0.97	0.99	-0.07	0.80	0.97	0.19	0.49	0.86
**Cobalamin**	0.08	0.78	0.98	-0.09	0.75	0.97	-0.02	0.94	0.99
**Niacin**	0.11	0.70	0.96	-0.09	0.73	0.97	0.07	0.79	0.97
**Animal-derived Protein**	-0.02	0.94	0.99	0.09	0.75	0.97	0.18	0.50	0.86
**Folic acid**	0.27	0.31	0.72	-0.08	0.77	0.97	0.03	0.90	0.98
**Glucose**	-0.06	0.83	0.98	0.08	0.76	0.97	0.34	0.20	0.63
**Plant-derived Protein**	0.35	0.19	0.63	-0.28	0.29	0.70	-0.31	0.25	0.67
**Fiber**	0.26	0.34	0.75	0.02	0.93	0.99	***<0.01***	0.99	1.00

Supplemental Table 7 (continued)

	g_Gemella			g_Clostridium_sensu_stricto_1			g_Family_XIII_AD3011_group		
	**Spearman Correlation**	***p* value**	***q* value**	**Spearman Correlation**	***p* value**	***q* value**	**Spearman Correlation**	***p* value**	***q* value**
**Tryptophan**	0.43	0.09	0.53	0.20	0.47	0.85	0.10	0.71	0.96
**Histidine**	0.45	0.08	0.50	0.18	0.52	0.87	0.09	0.74	0.97
**Lysine**	0.43	0.09	0.53	0.21	0.44	0.83	0.08	0.77	0.98
**Methionine**	0.39	0.14	0.56	0.34	0.20	0.63	0.09	0.75	0.97
**Lignoceric acid**	0.21	0.44	0.83	-0.05	0.86	0.98	-0.03	0.91	0.99
**Docosahexaenoic Acid**	0.22	0.42	0.83	0.23	0.40	0.81	0.12	0.67	0.94
**Clupanodonic acid**	0.15	0.57	0.90	0.15	0.58	0.91	0.24	0.38	0.79
**Arachidonic acid**	0.30	0.26	0.66	0.32	0.23	0.66	0.17	0.53	0.88
**Erucic acid**	0.49	0.05	0.46	-0.05	0.87	0.98	0.04	0.89	0.98
**Gadoleic acid**	0.30	0.26	0.66	0.14	0.60	0.91	0.13	0.62	0.92
**Palmitic acid**	0.27	0.31	0.72	0.25	0.35	0.77	0.22	0.40	0.81
**Margaric acid**	0.26	0.32	0.74	0.18	0.50	0.86	0.07	0.79	0.97
**Pentadecanoic acid**	0.12	0.66	0.94	0.27	0.30	0.72	-0.06	0.82	0.98
**Cholesterol**	0.34	0.20	0.62	0.42	0.11	0.55	0.10	0.72	0.96
**Cobalamin**	0.32	0.23	0.66	0.37	0.16	0.59	0.13	0.63	0.93
**Niacin**	0.35	0.19	0.63	0.28	0.30	0.71	0.34	0.20	0.63
**Animal-derived Protein**	0.31	0.24	0.66	0.24	0.37	0.79	0.24	0.37	0.79
**Folic acid**	0.09	0.74	0.97	0.21	0.44	0.83	-0.21	0.44	0.83
**Glucose**	-0.55	***0.03***	0.42	-0.07	0.80	0.97	0.03	0.91	0.99
**Plant-derived Protein**	0.07	0.81	0.97	-0.14	0.60	0.91	-0.21	0.44	0.83
**Fiber**	0.08	0.77	0.97	-0.06	0.82	0.98	-0.06	0.83	0.98

Supplemental Table 7 (continued)

	g_[Eubacterium]_eligens_group			g_[Eubacterium]_ventriosum_group			g_Anaerostipes		
	**Spearman Correlation**	***p* value**	***q* value**	**Spearman Correlation**	***p* value**	***q* value**	**Spearman Correlation**	***p* value**	***q* value**
**Tryptophan**	0.42	0.10	0.53	0.13	0.63	0.93	0.45	0.08	0.50
**Histidine**	0.35	0.19	0.63	0.08	0.76	0.97	0.33	0.22	0.65
**Lysine**	0.35	0.19	0.63	-0.05	0.85	0.98	0.31	0.25	0.66
**Methionine**	0.20	0.46	0.84	-0.04	0.89	0.98	0.22	0.42	0.83
**Lignoceric acid**	0.52	***0.04***	0.44	0.48	0.06	0.46	0.40	0.12	0.57
**Docosahexaenoic Acid**	-0.18	0.50	0.86	-0.34	0.20	0.63	-0.17	0.53	0.88
**Clupanodonic acid**	-0.15	0.57	0.91	-0.26	0.34	0.75	-0.11	0.68	0.95
**Arachidonic acid**	-0.21	0.43	0.83	-0.30	0.27	0.67	-0.11	0.69	0.96
**Erucic acid**	0.03	0.91	0.98	-0.20	0.45	0.83	0.09	0.75	0.97
**Gadoleic acid**	0.09	0.75	0.97	-0.02	0.94	0.99	0.14	0.61	0.92
**Palmitic acid**	0.07	0.78	0.98	-0.22	0.42	0.82	0.08	0.78	0.98
**Margaric acid**	0.14	0.60	0.91	-0.17	0.54	0.88	-0.04	0.88	0.98
**Pentadecanoic acid**	0.10	0.70	0.96	-0.05	0.84	0.98	0.06	0.83	0.98
**Cholesterol**	-0.15	0.59	0.91	-0.31	0.24	0.67	-0.10	0.71	0.96
**Cobalamin**	-0.15	0.59	0.91	-0.38	0.14	0.56	-0.12	0.66	0.94
**Niacin**	0.43	0.09	0.53	0.02	0.95	0.99	0.42	0.11	0.54
**Animal-derived Protein**	-0.01	0.98	1.00	-0.15	0.57	0.90	-0.04	0.87	0.99
**Folic acid**	0.19	0.48	0.86	0.01	0.96	0.99	0.28	0.29	0.70
**Glucose**	0.05	0.85	0.98	0.25	0.36	0.77	-0.07	0.79	0.97
**Plant-derived Protein**	0.33	0.22	0.65	0.27	0.32	0.73	0.30	0.25	0.66
**Fiber**	0.42	0.10	0.52	0.51	***0.04***	0.44	0.39	0.13	0.57

Supplemental Table 7 (continued)

	g_Dorea			g_Lachnoclostridium			g_Lachnospiraceae_ND3007_group		
	Spearman Correlation	*p* value	*q* value	Spearman Correlation	*p* value	*q* value	Spearman Correlation	*p* value	*q* value
**Tryptophan**	0.20	0.46	0.84	0.52	***0.04***	0.44	0.15	0.59	0.91
**Histidine**	0.08	0.77	0.97	0.63	***0.01***	0.29	0.07	0.80	0.97
**Lysine**	0.03	0.92	0.98	0.56	***0.02***	0.42	0.03	0.90	0.98
**Methionine**	0.13	0.62	0.92	0.52	***0.04***	0.43	0.06	0.82	0.98
**Lignoceric acid**	-0.06	0.82	0.98	-0.12	0.66	0.94	0.16	0.55	0.90
**Docosahexaenoic Acid**	0.11	0.68	0.96	0.38	0.14	0.56	-0.02	0.94	0.99
**Clupanodonic acid**	0.24	0.38	0.79	0.35	0.19	0.63	0.04	0.88	0.98
**Arachidonic acid**	0.16	0.56	0.90	0.35	0.18	0.62	***<0.01***	1.00	1.00
**Erucic acid**	0.14	0.60	0.91	0.62	***0.01***	0.28	-0.10	0.71	0.96
**Gadoleic acid**	0.15	0.58	0.91	0.47	0.07	0.49	0.05	0.85	0.98
**Palmitic acid**	0.26	0.33	0.74	0.55	***0.03***	0.43	0.14	0.60	0.91
**Margaric acid**	0.06	0.83	0.98	0.48	0.06	0.48	-0.03	0.92	0.98
**Pentadecanoic acid**	0.06	0.82	0.98	0.48	0.06	0.48	-0.03	0.91	0.99
**Cholesterol**	0.14	0.61	0.92	0.33	0.22	0.65	-0.01	0.97	0.99
**Cobalamin**	-0.03	0.92	0.98	0.43	0.10	0.52	-0.14	0.60	0.91
**Niacin**	0.23	0.40	0.81	0.54	***0.03***	0.43	0.28	0.29	0.70
**Animal-derived Protein**	0.12	0.67	0.94	0.65	***0.01***	0.44	0.05	0.85	0.98
**Folic acid**	-0.10	0.71	0.96	-0.32	0.23	0.66	-0.06	0.83	0.98
**Glucose**	***<0.01***	0.99	1.00	-0.31	0.25	0.66	0.34	0.20	0.63
**Plant-derived Protein**	-0.03	0.91	0.99	-0.55	***0.03***	0.42	0.02	0.93	0.99
**Fiber**	0.09	0.74	0.97	-0.38	0.15	0.57	0.21	0.45	0.83

Supplemental Table 7 (continued)

	g_Lactonifactor			g_Marvinbryantia			g_Intestinibacter		
	**Spearman Correlation**	***p* value**	***q* value**	**Spearman Correlation**	***p* value**	***q* value**	**Spearman Correlation**	***p* value**	***q* value**
**Tryptophan**	0.37	0.15	0.57	0.41	0.12	0.56	0.24	0.37	0.79
**Histidine**	0.44	0.09	0.51	0.36	0.17	0.60	0.19	0.49	0.85
**Lysine**	0.39	0.13	0.56	0.38	0.15	0.57	0.19	0.48	0.86
**Methionine**	0.41	0.12	0.56	0.36	0.17	0.59	0.04	0.89	0.98
**Lignoceric acid**	0.27	0.32	0.73	-0.09	0.73	0.97	0.36	0.18	0.62
**Docosahexaenoic Acid**	0.26	0.32	0.74	0.35	0.18	0.62	-0.23	0.39	0.81
**Clupanodonic acid**	0.33	0.22	0.65	0.39	0.13	0.56	-0.23	0.40	0.81
**Arachidonic acid**	0.30	0.26	0.66	0.40	0.12	0.57	-0.22	0.40	0.81
**Erucic acid**	0.09	0.73	0.97	0.45	0.08	0.50	0.01	0.98	1.00
**Gadoleic acid**	0.45	0.08	0.51	0.38	0.14	0.56	0.01	0.96	0.99
**Palmitic acid**	0.16	0.56	0.90	0.50	***0.05***	0.45	-0.09	0.75	0.97
**Margaric acid**	0.31	0.25	0.67	0.35	0.18	0.63	-0.10	0.70	0.96
**Pentadecanoic acid**	0.25	0.36	0.77	0.19	0.47	0.85	0.14	0.62	0.92
**Cholesterol**	0.20	0.46	0.84	0.34	0.19	0.63	-0.26	0.33	0.74
**Cobalamin**	0.40	0.12	0.57	0.30	0.26	0.66	-0.15	0.57	0.90
**Niacin**	0.30	0.26	0.66	0.53	***0.03***	0.42	0.22	0.41	0.82
**Animal-derived Protein**	0.40	0.12	0.57	0.33	0.21	0.65	-0.19	0.48	0.86
**Folic acid**	-0.17	0.52	0.88	0.04	0.88	0.99	0.38	0.15	0.57
**Glucose**	-0.23	0.39	0.81	-0.33	0.21	0.64	0.01	0.98	1.00
**Plant-derived Protein**	-0.09	0.73	0.97	-0.11	0.69	0.96	0.24	0.37	0.79
**Fiber**	-0.19	0.49	0.85	-0.13	0.63	0.93	0.18	0.51	0.86

Supplemental Table 7 (continued)

	g_Romboutsia			g_[Eubacterium]_coprostanoligenes_group			g_Faecalibacterium		
	Spearman Correlation	*p* value	*q* value	Spearman Correlation	*p* value	*q* value	Spearman Correlation	*p* value	*q* value
**Tryptophan**	0.65	0.01	0.41	-0.08	0.77	0.97	0.11	0.69	0.96
**Histidine**	0.64	0.01	0.32	-0.06	0.82	0.98	0.09	0.73	0.97
**Lysine**	0.68	***<0.01***	0.41	-0.16	0.57	0.90	-0.03	0.90	0.98
**Methionine**	0.64	***0.01***	0.33	0.05	0.86	0.98	0.04	0.88	0.99
**Lignoceric acid**	0.01	0.96	0.99	-0.01	0.99	1.00	0.38	0.15	0.57
**Docosahexaenoic Acid**	0.44	0.09	0.52	0.11	0.69	0.96	-0.20	0.46	0.84
**Clupanodonic acid**	0.35	0.19	0.63	0.23	0.38	0.80	-0.13	0.63	0.93
**Arachidonic acid**	0.51	***0.04***	0.44	0.14	0.60	0.91	-0.16	0.55	0.90
**Erucic acid**	0.38	0.14	0.56	-0.35	0.18	0.62	-0.25	0.35	0.77
**Gadoleic acid**	0.51	***0.04***	0.44	0.05	0.84	0.98	-0.07	0.80	0.97
**Palmitic acid**	0.60	***0.01***	0.31	0.02	0.94	0.99	-0.09	0.75	0.97
**Margaric acid**	0.41	0.12	0.56	0.11	0.68	0.95	-0.02	0.94	0.99
**Pentadecanoic acid**	0.28	0.29	0.70	0.12	0.66	0.94	0.06	0.82	0.98
**Cholesterol**	0.52	***0.04***	0.43	0.11	0.69	0.96	-0.09	0.73	0.97
**Cobalamin**	0.45	0.08	0.51	0.07	0.78	0.97	-0.15	0.57	0.90
**Niacin**	0.72	***<0.01***	0.24	-0.08	0.78	0.98	0.09	0.75	0.97
**Animal-derived Protein**	0.50	***0.05***	0.45	0.18	0.51	0.86	0.04	0.89	0.98
**Folic acid**	0.19	0.49	0.85	-0.31	0.24	0.66	-0.09	0.74	0.97
**Glucose**	-0.18	0.51	0.86	0.21	0.44	0.83	0.37	0.16	0.57
**Plant-derived Protein**	-0.10	0.70	0.96	-0.15	0.59	0.91	0.12	0.66	0.94
**Fiber**	-0.05	0.85	0.98	-0.02	0.94	0.99	0.39	0.14	0.56

Supplemental Table 7 (continued)

	g_Ruminiclostridium_5			g_Ruminiclostridium_9			g_Ruminococcaceae_UCG_014		
	Spearman Correlation	*p* value	*q* value	Spearman Correlation	*p* value	*q* value	Spearman Correlation	*p* value	*q* value
**Tryptophan**	0.62	0.01	0.29	0.45	0.08	0.50	-0.02	0.93	0.99
**Histidine**	0.67	***<0.01***	0.41	0.63	0.01	0.28	-0.07	0.79	0.97
**Lysine**	0.58	***0.02***	0.36	0.55	***0.03***	0.42	-0.08	0.77	0.98
**Methionine**	0.63	***0.01***	0.27	0.53	***0.03***	0.43	-0.10	0.72	0.97
**Lignoceric acid**	0.02	0.94	0.99	-0.23	0.40	0.81	-0.06	0.81	0.98
**Docosahexaenoic Acid**	0.43	0.10	0.52	0.36	0.18	0.62	-0.13	0.64	0.93
**Clupanodonic acid**	0.49	0.05	0.45	0.39	0.14	0.56	0.04	0.90	0.98
**Arachidonic acid**	0.43	0.10	0.52	0.31	0.25	0.67	-0.12	0.67	0.94
**Erucic acid**	0.31	0.23	0.66	0.49	0.06	0.46	-0.07	0.80	0.98
**Gadoleic acid**	0.58	***0.02***	0.36	0.47	0.07	0.48	-0.10	0.71	0.96
**Palmitic acid**	0.49	0.06	0.45	0.49	0.05	0.46	0.14	0.60	0.91
**Margaric acid**	0.62	***0.01***	0.28	0.75	***<0.01***	0.18	-0.10	0.72	0.97
**Pentadecanoic acid**	0.55	***0.03***	0.42	0.78	***<0.01***	0.14	-0.06	0.83	0.98
**Cholesterol**	0.34	0.20	0.63	0.28	0.29	0.70	-0.09	0.73	0.97
**Cobalamin**	0.53	***0.03***	0.43	0.54	***0.03***	0.43	-0.13	0.62	0.93
**Niacin**	0.55	***0.03***	0.43	0.39	0.13	0.56	0.27	0.32	0.73
**Animal-derived Protein**	0.63	***0.01***	0.28	0.58	***0.02***	0.37	0.03	0.91	0.98
**Folic acid**	-0.29	0.28	0.69	-0.06	0.83	0.98	-0.05	0.86	0.98
**Glucose**	-0.33	0.21	0.63	-0.47	0.07	0.48	0.40	0.13	0.57
**Plant-derived Protein**	-0.30	0.26	0.66	-0.47	0.07	0.48	-0.16	0.56	0.90
**Fiber**	-0.30	0.25	0.66	-0.43	0.09	0.53	0.11	0.69	0.96

Supplemental Table 7 (continued)

	g_Coprobacillus			g_Erysipelatoclostridium			g_Erysipelotrichaceae_UCG_003		
	Spearman Correlation	*p* value	*q* value	Spearman Correlation	*p* value	*q* value	Spearman Correlation	*p* value	*q* value
**Tryptophan**	0.41	0.11	0.56	0.13	0.63	0.93	0.28	0.30	0.71
**Histidine**	0.52	0.04	0.43	0.06	0.83	0.98	0.35	0.18	0.62
**Lysine**	0.46	0.07	0.50	0.12	0.67	0.94	0.25	0.35	0.77
**Methionine**	0.48	0.06	0.47	0.26	0.34	0.75	0.12	0.67	0.94
**Lignoceric acid**	0.29	0.27	0.67	-0.26	0.33	0.73	0.43	0.09	0.53
**Docosahexaenoic Acid**	0.35	0.18	0.63	0.32	0.22	0.65	-0.17	0.52	0.88
**Clupanodonic acid**	0.32	0.23	0.66	0.21	0.44	0.83	-0.19	0.49	0.85
**Arachidonic acid**	0.39	0.14	0.56	0.35	0.18	0.62	-0.12	0.65	0.94
**Erucic acid**	0.21	0.44	0.83	-0.22	0.41	0.82	0.18	0.49	0.85
**Gadoleic acid**	0.49	0.06	0.46	0.07	0.79	0.97	0.12	0.65	0.94
**Palmitic acid**	0.19	0.48	0.86	0.17	0.53	0.88	0.04	0.87	0.99
**Margaric acid**	0.47	0.07	0.49	-0.05	0.86	0.98	0.21	0.43	0.83
**Pentadecanoic acid**	0.39	0.14	0.56	0.08	0.77	0.97	0.16	0.55	0.90
**Cholesterol**	0.31	0.24	0.66	0.35	0.19	0.63	-0.14	0.60	0.91
**Cobalamin**	0.50	***0.05***	0.45	0.42	0.11	0.53	-0.13	0.63	0.92
**Niacin**	0.28	0.29	0.70	0.17	0.53	0.87	0.30	0.26	0.66
**Animal-derived Protein**	0.40	0.13	0.56	0.24	0.36	0.78	***<0.01***	0.99	1.00
**Folic acid**	***<0.01***	1.00	1.00	-0.14	0.60	0.91	0.30	0.25	0.66
**Glucose**	-0.31	0.25	0.66	0.17	0.52	0.87	-0.02	0.96	0.99
**Plant-derived Protein**	***<0.01***	1.00	1.00	-0.23	0.39	0.81	0.21	0.43	0.83
**Fiber**	-0.20	0.46	0.84	-0.37	0.16	0.58	0.39	0.14	0.56

Supplemental Table 7 (continued)

	g_Holdemanella			g_Holdemania			g_Turicibacter		
	**Spearman Correlation**	***p* value**	***q* value**	**Spearman Correlation**	***p* value**	***q* value**	**Spearman Correlation**	***p* value**	***q* value**
**Tryptophan**	0.31	0.24	0.66	0.42	0.10	0.53	0.31	0.24	0.66
**Histidine**	0.21	0.43	0.83	0.58	0.02	0.35	0.31	0.24	0.67
**Lysine**	0.26	0.33	0.74	0.51	***0.04***	0.44	0.37	0.15	0.57
**Methionine**	0.40	0.13	0.57	0.49	0.05	0.46	0.18	0.52	0.87
**Lignoceric acid**	-0.29	0.27	0.67	-0.04	0.88	0.98	0.04	0.89	0.98
**Docosahexaenoic Acid**	0.48	0.06	0.46	0.38	0.15	0.56	-0.09	0.75	0.97
**Clupanodonic acid**	0.39	0.14	0.56	0.28	0.30	0.72	-0.24	0.38	0.79
**Arachidonic acid**	0.54	***0.03***	0.43	0.44	0.09	0.51	-0.03	0.91	0.99
**Erucic acid**	0.12	0.65	0.94	0.22	0.42	0.82	0.14	0.61	0.92
**Gadoleic acid**	0.31	0.24	0.66	0.47	0.07	0.48	0.02	0.95	0.99
**Palmitic acid**	0.47	0.06	0.48	0.34	0.19	0.63	0.21	0.44	0.83
**Margaric acid**	0.19	0.48	0.85	0.56	***0.02***	0.42	0.08	0.76	0.97
**Pentadecanoic acid**	0.03	0.90	0.98	0.43	0.10	0.53	0.05	0.85	0.98
**Cholesterol**	0.54	***0.03***	0.43	0.34	0.20	0.63	0.03	0.92	0.98
**Cobalamin**	0.36	0.17	0.61	0.44	0.08	0.51	-0.03	0.91	0.99
**Niacin**	0.40	0.13	0.56	0.31	0.25	0.66	0.43	0.10	0.52
**Animal-derived Protein**	0.31	0.24	0.67	0.40	0.13	0.56	-0.05	0.84	0.98
**Folic acid**	0.01	0.98	1.00	0.20	0.45	0.83	0.63	***0.01***	0.27
**Glucose**	0.19	0.49	0.86	-0.26	0.33	0.74	***<0.01***	0.99	1.00
**Plant-derived Protein**	-0.07	0.81	0.98	-0.06	0.84	0.98	0.20	0.45	0.83
**Fiber**	-0.15	0.59	0.91	-0.14	0.59	0.91	0.32	0.23	0.66

Supplemental Table 7 (continued)

	**g_Phascolarctobacterium**			**g_Bilophila**			**g_Enterobacter**		
	**Spearman Correlation**	***p* value**	***q* value**	**Spearman Correlation**	***p* value**	***q* value**	**Spearman Correlation**	***p* value**	***q* value**
**Tryptophan**	0.20	0.45	0.83	0.15	0.58	0.91	0.53	0.04	0.43
**Histidine**	0.33	0.21	0.64	0.26	0.33	0.74	0.37	0.16	0.57
**Lysine**	0.22	0.41	0.82	0.22	0.41	0.82	0.43	0.10	0.52
**Methionine**	0.15	0.57	0.90	0.32	0.22	0.65	0.33	0.21	0.64
**Lignoceric acid**	-0.10	0.72	0.97	-0.47	0.07	0.47	0.02	0.94	0.99
**Docosahexaenoic Acid**	0.04	0.88	0.99	0.51	*0.04*	0.44	0.19	0.48	0.85
**Clupanodonic acid**	0.05	0.85	0.98	0.45	0.08	0.50	0.10	0.70	0.96
**Arachidonic acid**	-0.02	0.95	0.99	0.44	0.08	0.51	0.16	0.56	0.90
**Erucic acid**	0.59	***0.02***	0.36	0.37	0.15	0.57	0.18	0.50	0.86
**Gadoleic acid**	0.09	0.74	0.97	0.24	0.37	0.79	0.34	0.20	0.63
**Palmitic acid**	0.30	0.26	0.66	0.43	0.10	0.52	0.13	0.63	0.93
**Margaric acid**	0.28	0.29	0.71	0.50	***0.05***	0.45	0.12	0.65	0.94
**Pentadecanoic acid**	0.47	0.07	0.48	0.53	***0.03***	0.43	0.07	0.79	0.97
**Cholesterol**	0.04	0.88	0.99	0.41	0.11	0.54	-0.01	0.96	0.99
**Cobalamin**	0.08	0.76	0.97	0.59	***0.02***	0.35	0.21	0.42	0.83
**Niacin**	0.19	0.48	0.86	0.16	0.55	0.90	0.39	0.14	0.56
**Animal-derived Protein**	0.32	0.22	0.65	0.53	***0.04***	0.42	-0.03	0.92	0.98
**Folic acid**	-0.16	0.56	0.90	-0.40	0.13	0.57	0.16	0.55	0.90
**Glucose**	-0.24	0.38	0.79	-0.29	0.28	0.70	-0.21	0.44	0.83
**Plant-derived Protein**	-0.44	0.09	0.51	-0.59	***0.02***	0.36	0.30	0.25	0.66
**Fiber**	-0.19	0.49	0.85	-0.72	***<0.01***	0.21	-0.18	0.51	0.86

Supplemental Table 7 (continued)

Species	s_Bacteroides_coprocola_DSM_17136			s_Bifidobacterium_longum_subsp_longum			s_Parabacteroides_merdae		
	Spearman Correlation	*p* value	*q* value	Spearman Correlation	*p* value	*q* value	Spearman Correlation	*p* value	*q* value
**Tryptophan**	-0.31	0.25	0.69	0.19	0.49	1.00	0.18	0.49	1.00
**Histidine**	-0.43	0.10	0.54	0.09	0.75	1.00	0.26	0.34	0.99
**Lysine**	-0.49	0.05	0.54	0.07	0.80	1.00	0.20	0.46	1.00
**Methionine**	-0.40	0.12	0.51	0.17	0.53	1.00	0.22	0.42	1.00
**Lignoceric acid**	0.08	0.77	0.93	0.04	0.90	1.00	0.10	0.71	1.00
**Docosahexaenoic Acid**	-0.21	0.44	0.75	0.09	0.73	1.00	-0.07	0.80	1.00
**Clupanodonic acid**	-0.35	0.19	0.64	0.05	0.86	1.00	-0.04	0.89	1.00
**Arachidonic acid**	-0.27	0.31	0.69	0.17	0.54	1.00	-0.10	0.73	1.00
**Erucic acid**	-0.22	0.42	0.73	0.07	0.78	1.00	0.05	0.84	1.00
**Gadoleic acid**	-0.49	0.06	0.51	-0.01	0.98	1.00	-0.04	0.88	1.00
**Palmitic acid**	-0.42	0.10	0.54	0.20	0.46	0.98	0.27	0.31	0.97
**Margaric acid**	-0.39	0.14	0.55	0.10	0.72	1.00	0.43	0.09	0.63
**Pentadecanoic acid**	-0.25	0.35	0.72	0.08	0.76	1.00	0.61	***0.01***	0.49
**Cholesterol**	-0.25	0.34	0.72	0.29	0.28	0.94	0.14	0.59	1.00
**Cobalamin**	-0.29	0.28	0.70	0.04	0.89	1.00	0.06	0.82	1.00
**Niacin**	-0.47	0.07	0.54	0.15	0.58	1.00	0.28	0.29	0.95
**Animal-derived Protein**	-0.41	0.12	0.56	0.03	0.91	1.00	0.17	0.53	1.00
**Folic acid**	-0.23	0.40	0.74	0.23	0.40	0.96	0.34	0.20	0.89
**Glucose**	0.28	0.30	0.70	-0.01	0.97	1.00	0.21	0.45	1.00
**Plant-derived Protein**	0.31	0.24	0.73	0.23	0.40	0.96	-0.09	0.74	1.00
**Fiber**	0.06	0.82	0.95	0.29	0.28	0.94	0.23	0.39	1.00

Supplemental Table 7 (continued)

	s_bacterium_NLAE_zl_C326			s_Actinomyces_odontolyticus			s_Streptococcus_mutans		
	**Spearman Correlation**	***p* value**	***q* value**	**Spearman Correlation**	***p* value**	***q* value**	**Spearman Correlation**	***p* value**	***q* value**
**Tryptophan**	0.37	0.16	0.88	0.53	0.04	0.57	0.13	0.64	1.00
**Histidine**	0.46	0.07	0.67	0.62	0.01	1.00	0.09	0.73	1.00
**Lysine**	0.50	0.05	0.63	0.59	***0.02***	0.70	0.07	0.79	1.00
**Methionine**	0.46	0.07	0.61	0.62	***0.01***	1.00	0.36	0.17	1.00
**Lignoceric acid**	0.09	0.75	1.00	0.24	0.37	1.00	-0.02	0.95	1.00
**Docosahexaenoic Acid**	0.26	0.34	1.00	0.33	0.21	1.00	0.40	0.12	1.00
**Clupanodonic acid**	0.21	0.42	1.00	0.28	0.29	1.00	0.29	0.28	1.00
**Arachidonic acid**	0.33	0.22	0.98	0.35	0.19	1.00	0.46	0.07	1.00
**Erucic acid**	0.15	0.58	1.00	0.11	0.69	1.00	-0.33	0.22	1.00
**Gadoleic acid**	0.30	0.26	1.00	0.45	0.08	0.92	0.17	0.53	1.00
**Palmitic acid**	0.36	0.17	0.91	0.30	0.26	1.00	0.14	0.60	1.00
**Margaric acid**	0.31	0.25	1.00	0.54	***0.03***	0.77	0.25	0.36	1.00
**Pentadecanoic acid**	0.20	0.46	1.00	0.48	0.06	0.83	0.23	0.39	1.00
**Cholesterol**	0.41	0.12	0.78	0.39	0.14	1.00	0.54	***0.03***	1.00
**Cobalamin**	0.46	0.07	0.61	0.55	***0.03***	0.89	0.41	0.12	1.00
**Niacin**	0.49	0.06	0.64	0.42	0.11	1.00	-0.01	0.97	1.00
**Animal-derived Protein**	0.52	***0.04***	0.53	0.54	***0.03***	0.58	0.23	0.39	1.00
**Folic acid**	***<0.01***	0.99	1.00	-0.06	0.84	1.00	0.02	0.95	1.00
**Glucose**	-0.17	0.53	1.00	-0.31	0.24	1.00	0.25	0.35	1.00
**Plant-derived Protein**	-0.27	0.31	1.00	-0.13	0.63	1.00	0.11	0.69	1.00
**Fiber**	-0.06	0.83	1.00	-0.12	0.65	1.00	-0.06	0.83	1.00
